# International prevalence of tactile map usage and its impact on navigational independence and well-being of people with visual impairments

**DOI:** 10.1038/s41598-025-08117-9

**Published:** 2025-07-26

**Authors:** Maxime Bleau, Kopila Kafle, Min Wang, Soutongnoma Safiata Kabore, Jorge Luis Cueva-Vargas, Joseph Paul Nemargut

**Affiliations:** 1 School of Optometry, Université de Montréal, Montreal, QC Canada; 2 Centre for Interdisciplinary Research in Rehabilitation of Greater Montreal (CRIR), Montreal, QC Canada; 3 Center for Interdisciplinary Research on Brain and Learning (CIRCA), Université de Montréal, Montreal, QC Canada; 4 Universidad Cesar Vallejo, Trujillo, La Libertad, Peru

**Keywords:** Human behaviour, Quality of life

## Abstract

For people with visual impairments (PVI), understanding space is crucial for independence and tactile maps are useful tools to gain spatial information and improve orientation. However, their popularity and impacts in the PVI population are not yet fully understood. Thus, this study aims to determine the prevalence of tactile map usage and their effects on independent travel and well-being internationally. To do so, two online surveys were completed by PVI (n_1_ = 752, n_2_ = 510) in 40 countries from which information was collected related to travel habits, spatial abilities, experience with tactile maps, mobility services, and perceived well-being. The surveys revealed that only 17.15% of respondents have had experience with tactile maps and that this tactile map experience was related to better cognitive mapping skills, a higher level of education, a higher perceived well-being and a higher sense of independence (i.e., perceiving the area of living as more accessible, and requiring assistance less often). This study confirms that tactile maps have a positive impact on the independent travel and well-being of PVI around the world, including both individuals with low-vision and blindness, and demonstrates the importance of early tactile map exposure, as tactile maps support the development of generalizable spatial concepts and abilities.

**Supplementary Information:**

The online version contains supplementary material available at 10.1038/s41598-025-08117-9.

## Introduction

Vision is the most adapted sense for humans to gather sensory information from the environment and, therefore, to move around and interact with it, visual impairments (VI) can have dire consequences on mobility and orientation abilities^1,2^, and thus on social and economic participation and quality of life^3–5^. Indeed, people with visual impairments (PVI) often experience higher cognitive and physical demands when navigating the world, especially when the environment is inaccessible, or the tools and strategies are not tailored to their needs^6,7^. As a result, PVI can face challenges when exploring unfamiliar environments and can limit themselves to familiar places or routes. This limitation primarily arises from the reliance on limited and less precise sensory information^8,9^, which impacts their ability to form and utilize cognitive maps of their surroundings^10,11^.

Cognitive map formation, or cognitive mapping, is a process that involves the creation of a mental representation of a physical environment that includes the positions and spatial relationships of all relevant objects, landmarks, and paths of the environment^12–14^. This ability is crucial for navigation, especially without visual input, as it facilitates orientation by improving awareness of relevant sensory information and one’s position and movement in space. Indeed, when having a complete cognitive map of their surroundings, PVI can relate it to the information they perceive while navigating and solving problematic situations (i.e., blocked paths, wrong turns).

To improve their cognitive mapping skills and navigational independence, PVI can access specialized mobility services, such as Orientation & Mobility (O&M) services, to learn how to use various strategies and tools to navigate safely and orient themselves more effectively^15^. Some studies have shown that tactile maps, traditionally made in embossed or Braille paper or handcrafted by rehabilitation professionals, are one of the most valuable, low-cost and versatile tools used in O&M to facilitate cognitive mapping^16–20^. With tactile maps, PVI can, within the span of two hands, access valuable “survey knowledge” of the environment, which includes all possible paths, directions, and destinations^21,22^. As a result of this, tactile map training, compared to other strategies, leads to more complete and precise cognitive maps, allowing PVI to learn their environment faster and even take shortcuts to the same destinations^23,24^. This can be possible by allowing the person to explore the environment in danger- and stress-free situations, to prepare their cognitive map before traveling in the real environment, and/or by allowing them to constantly relate what they touch to what they experience while traveling^23,25–29^.

However, tactile maps can be a challenging tool to learn for PVI^19,30^. They require many underlying abilities^31^, such as (1) *improved tactile sensitivity*^32^; (2) *knowledge of 2D line drawing rules* (in the case of traditional paper maps)^31–34^; (3) *working memory* abilities to integrate large amounts of information (memory load)^18^; (4) the ability to use *cardinal directions*; (5) *environmental scaling*, or the ability to relate a small-scale model to a real environment^31,35^; (6) *mental navigation*, or the ability to imagine oneself moving in an environment without proprioceptive and vestibular feedback^36^; and (7) *mental rotation*, or the ability to mentally change one’s point of view^32,37,38^. These spatial cognition skills can also be challenging to develop with limited or no access to visual information^14^, even more so for those with early onset VI since these typically develop between 7 and 10 years of age^39–41^. Furthermore, tactile maps provide fewer sensory details than direct exploration and, consequentially, can become more abstract or even senseless for some users^19^. This contributes to the difficulty of adopting these tools, and some PVI still prefer to learn the environment directly rather than learn to use tactile maps to their full potential.

Though tactile maps have been shown to improve spatial cognition and orientation, no prior studies, to our knowledge, have explored the prevalence or popularity of tactile maps in combination with their impact on the independence and quality of life of PVI. Consequently, their benefits within or outside mobility services (i.e., O&M or specialized schools) remain unclear, as are their related teaching strategies and other sociodemographic factors contributing to their adoption and success. Therefore, the present study aims to determine, for the first time, the worldwide prevalence of tactile map usage with a specific emphasis on their impact on independent travel and the well-being of PVI. Through two international surveys of the PVI population from 6 different continents, this study paints a broad portrait of PVI, the characteristics of their visual conditions, their life habits, their perceived independence, and their general sense of well-being. This data provides insights into the factors that enhance personal independence, self-actualization, and fulfillment in the PVI population, including the relative contribution of tactile maps and the context and goals for their use.

## Results

Out of 1437 entries, 752 PVI (410 M, 341 F, 1 N/A, mean age = 36.44 y; 47.67% attrition rate^42^) completed the first survey, which investigated demographic factors, life and travel habits, cognitive mapping skills, and the use of tactile maps. Out of these 752 respondents, 510 also completed survey 2 (32.18% attrition rate), which investigated specialized mobility training, well-being outcomes (as defined by the OMO tool part B^43^), and confidence in indoor and outdoor environments. On average, survey 1 took 30.33 min to complete, and survey 2 took 18.74 min. Detailed information regarding all the performed analyses and the tested variables can be found in supplementary file 2.

Demographics.

**Fig. 1:**
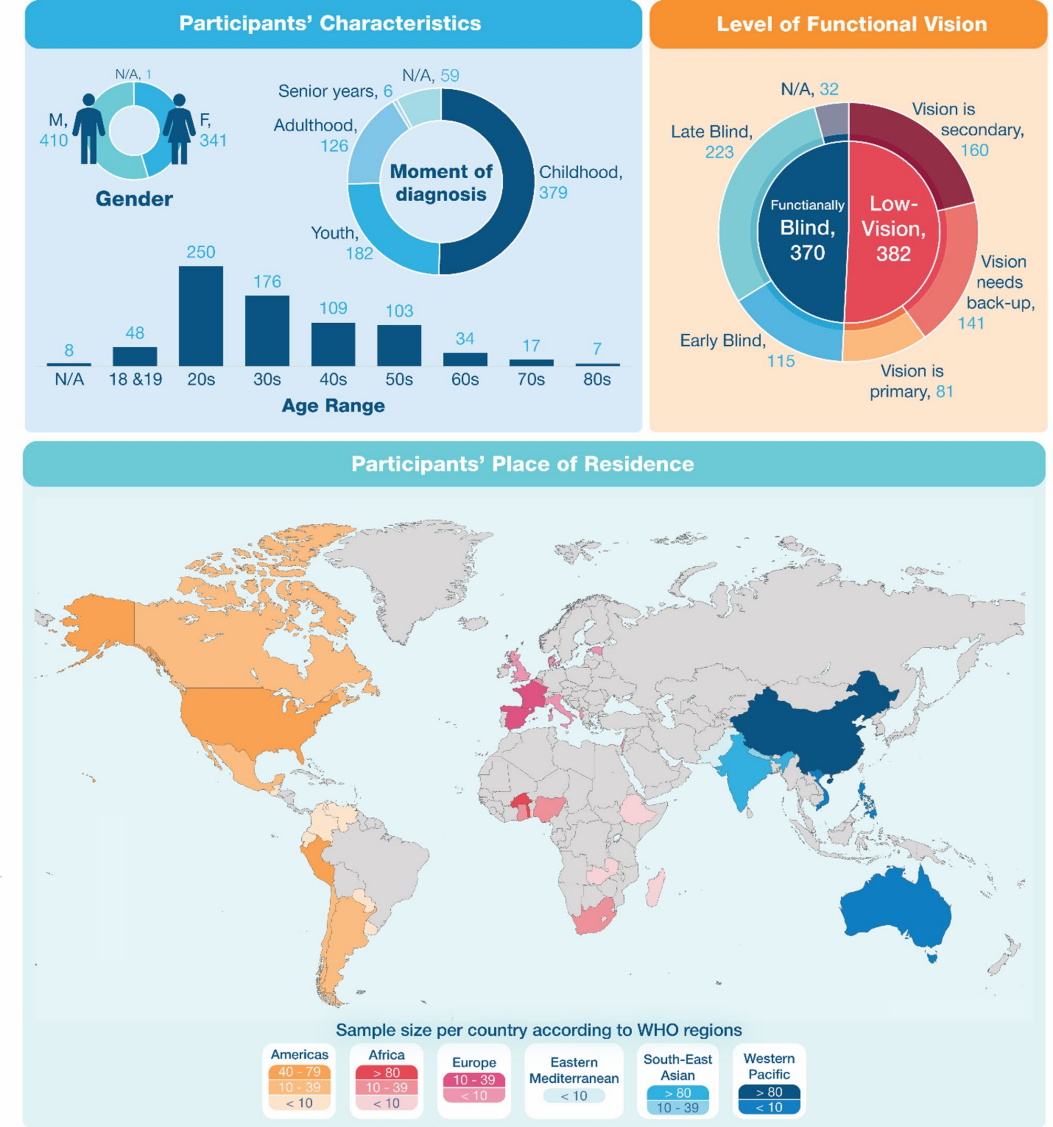
Sociodemographic profiles of participants, including gender, age category, moment when their visual condition was diagnosed, categorized according to the four age categories: childhood [0–14 years], youth [15–24 years], adulthood [25–64 years], senior years [65 years and over] (from https://www.statcan.gc.ca/en/concepts/definitions/age2);, their level of functional vision (including early blind [blind before six years] and late blind [blind after six years]; from VROOM tool^43^) and their country of living, categorized according to the six WHO world regions (from https://www.who.int/countries).

Participants were from 40 different countries within five WHO regions (224 participants from the Americas, 57 from Europe, 278 from Africa, 101 from South-East Asia; 92 from the Western Pacific region). Most participants declared living in urban areas (*n* = 444), while the others lived either in suburban (*n* = 167) or rural (*n* = 142) areas. In general, most participants graduated from an undergraduate university program (*n* = 280), or from high school (*n* = 162), and were from lower (*n* = 402) or middle (*n* = 342) economic classes (in the context of their region). Figure 1 illustrates the sociodemographic profiles of all participants.

In terms of visual condition, participants were, in average, diagnosed at the age of 17.69 years and the most common conditions were diabetic retinopathy (*n* = 185), glaucoma (*n* = 145), enucleation (*n* = 108), and cataracts (*n* = 85). The VROOM tool^43^ classification of visual functions was used to describe participants’ level of functional vision (e.g., how vision is used during navigation). Following this classification, most participants (*n* = 370) described their vision as being useless for navigation (in other words, functionally blind), as secondary (*n* = 160), or as needing backup from non-visual aids (*n* = 141), while only 81 did not need any non-visual aids or strategy to compensate for their vision loss. For those who identified as blind, the mean age at blindness onset was 16.82 years, totaling 115 early blind (EB; blind before six years old) and 223 late blind participants (LB; blind after six years old).

### Tactile maps usage: prevalence and user profiles

**Fig. 2:**
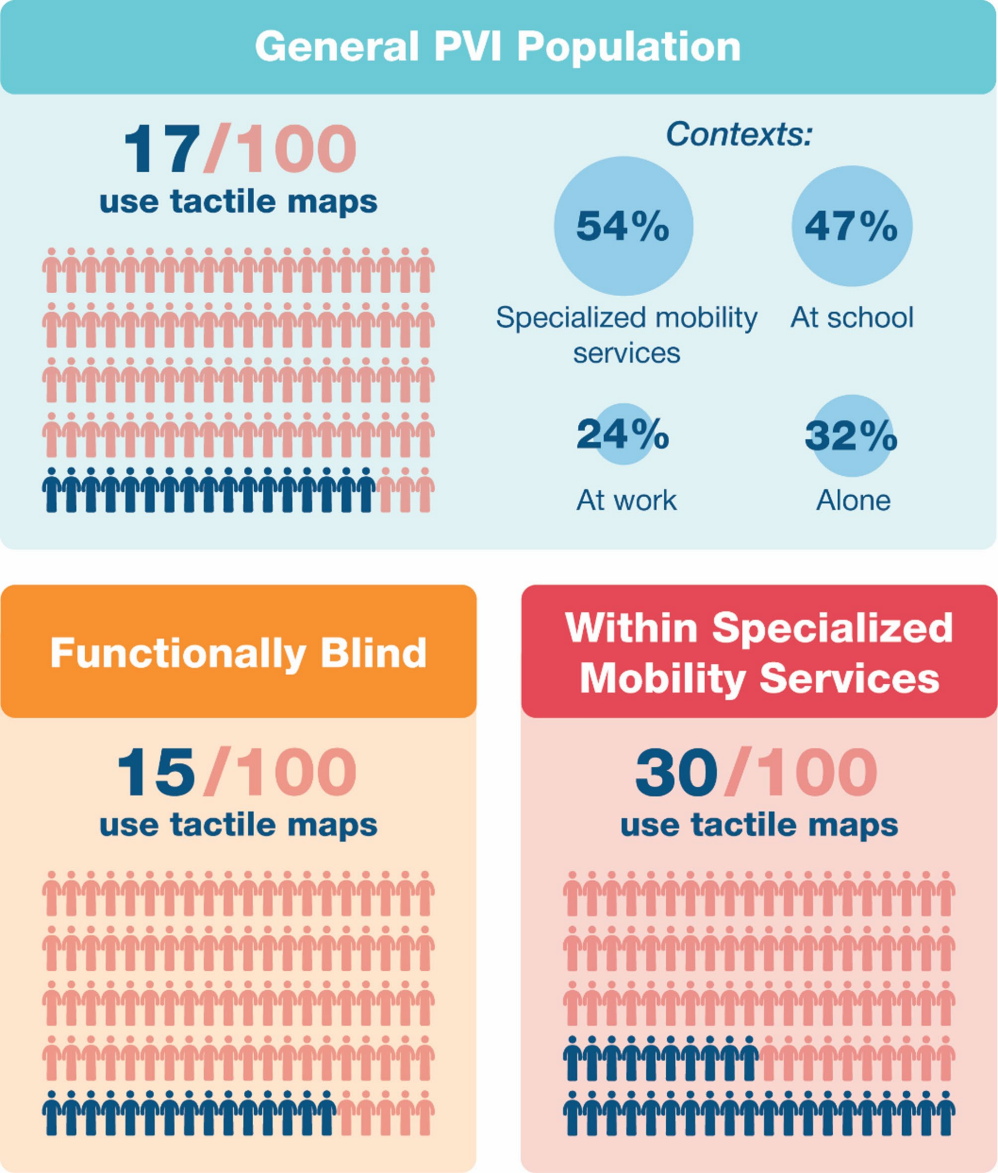
Prevalence of tactile map experience in the general PVI population (survey 1), in functionally blind participants (survey 1) and PVI who received specialized mobility services (survey 2) and the context of their usage.

Only 17.15% (*n* = 129/752) of respondents have had experience with tactile maps and their prevalence differed according to different regions of the world (X^2^(4,*n* = 748) = 68.129, *p* < .001): participants from the Americas and Europe were more likely to have this experience than participants from Africa and the Western Pacific. However, participants from urban, suburban and rural areas were, overall, as likely as each other to get experience with tactile maps (X^2^(2, *n* = 752) = 0.979, *p* = .616). Furthermore, tactile map users were no different from other PVI in terms of their level of visual function (1.98 vs. 1.88, U = 38093.5, *p* = .380), but tended to have their visual condition diagnosed earlier in life (9.78y vs. 19.25y; U = 43594, *p* < .001). Some contexts to use a tactile map were more common than others (Q(5) = 22.179, *p* < .001); the most common were within mobility services (*n* = 70) or at school (*n* = 60), and the least common were at work (*n* = 31) and alone (*n* = 41). Consistently with this result, those who received specialized mobility services were more likely to have had experience with tactile maps (57/187 vs. 23/323; X^2^(2, *n* = 510) = 47.117, *p* < .001). Figure 2 illustrates the prevalence and context of tactile maps usage.

Since tactile maps are produced in various ways (i.e., handcrafted or printed on Braille paper), the analysis investigated the different types of tactile maps and revealed that some were more common than others (Q(5) = 66.579, *p* < .001): the most common types were those made of Braille paper (*n* = 67), of textured (embossed) paper (*n* = 51) or handcrafted/glued (*n* = 50), while the least common were made from multiple materials (*n* = 33), from metal (*n* = 20) or handcrafted with Velcro (i.e., Picture Maker kit [https://www.aph.org/product/picture-maker-wheatley-tactile-diagramming-kit/]; *n* = 20). Finally, some categories of environments were more commonly represented than others (Q(2) = 6.742, *p* = .034): the most common categories were city maps (*n* = 80), followed by geographical maps (*n* = 67) and floor plans (*n* = 63). Around half of these participants used tactile maps to learn an environment (*n* = 65/129), of which 46 used them to develop a general understanding of different roads and addresses (outdoor environments); 44, to learn and practice specific routes; 36, to learn the layout of a building or room (indoor environments); and 34, to learn the layout of intersections for street crossing. The difference between these options was insignificant (Q(3) = 7.710, *p* = .052). Finally, only 58 of these participants had been exposed to publicly displayed tactile maps, but this was mostly occasional exposition (*n* = 46), while regular (*n* = 7) and daily (*n* = 5) exposition was less common. The analyses did not detect any significant benefits of this exposition.

### General impacts of tactile maps

**Fig. 3:**
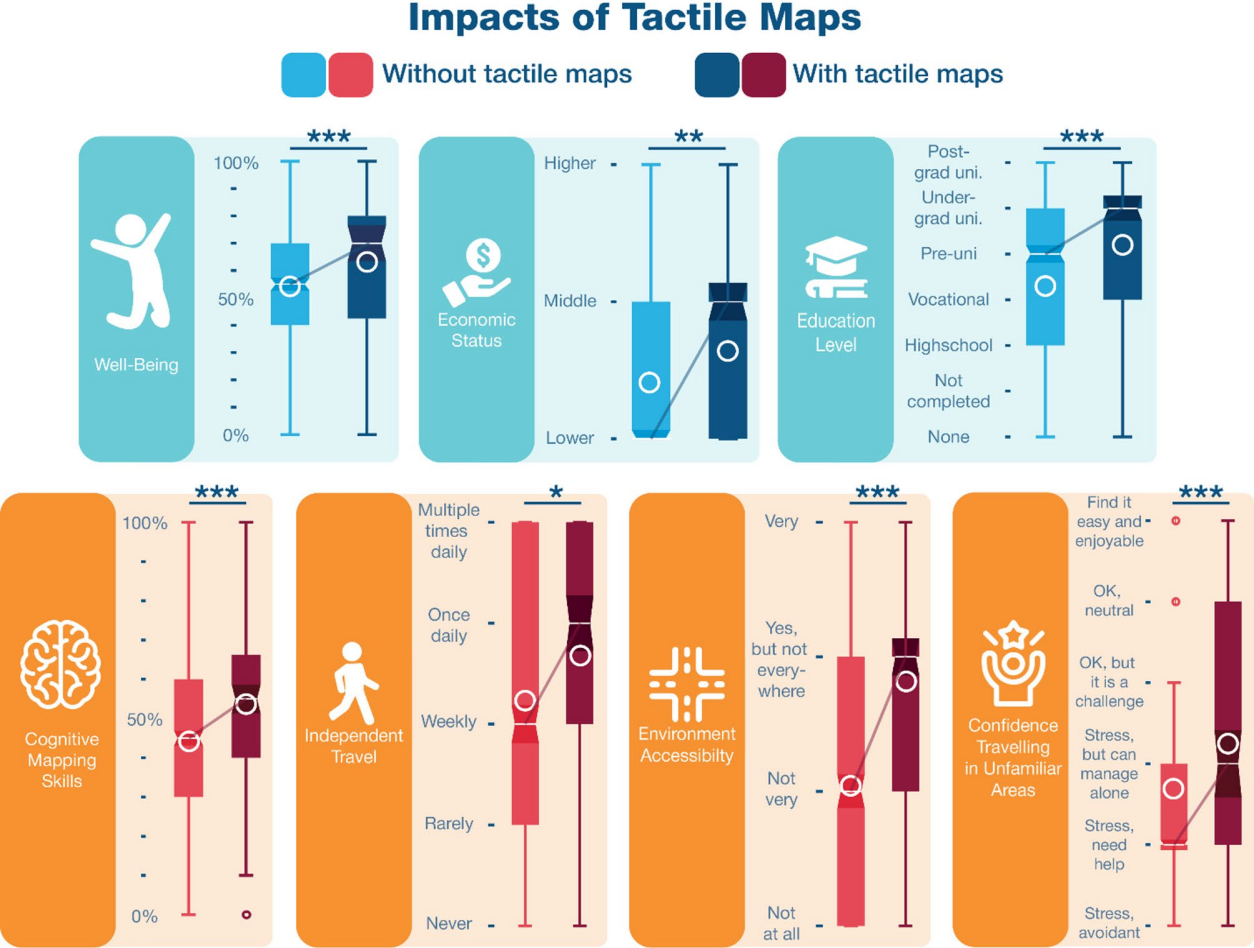
Impacts of tactile maps in the general PVI population. Whisker plots display the differences between tactile map users (darker shades of blue and red) and non-users (lighter shades of blue and red), including data medians (white bars) and means (white circles), notches illustrate the confidence interval around the median. Blue graphs refer to variables related to well-being and red graphs, to variables related to mobility and independence. The well-being score (0–100%) reflects the quality, or level, of engagement in activities, personal connections, orientation capacities, and life space, while the cognitive mapping skills score (0–100%) reflects participants’ ability and confidence to learn the layout of new environments. Icons from Freepik (https://www.freepik.com). *N.s.* nonsignificant; *p < .05; **p < .01; ***p < ..001.

Overall, respondents with tactile map experience appear to be more active travelers than non-tactile map users. Indeed, they travel more often both independently (3.67 vs. 3.36, U = 35082.5, *p* = .028, *r* = .086) and while accompanied (2.99 vs. 2.61, U = 31792, *p* < .001, *r* = .146), they travel in unfamiliar areas more frequently (2.40 vs. 2.19, U = 35012.5, *p* = .006, *r* = .147) and tended to travel for more reasons (i.e., school, work, groceries, shopping, medical appointments, leisure, exercise; 3.49 vs. 3.10, U = 35707, *p* = .060, n.s. trend, *r* = .074). Tactile maps usage was also related to general navigational skills, as tactile map users perceive their environment as more accessible (2.80 vs. 2.03, U = 22901, *p* < .001, *r* = .293), have better cognitive mapping skills (53.53% vs. 44.12%, U = 30827, *p* < .001, *r* = .152) and are more confident travelling in unfamiliar areas (3.25 vs. 2.69, U = 31684, *p* < .001, *r* = .147). Furthermore, tactile map usage was found to be related to a higher level of education completed (5.15 vs. 4.40, U = 38684, *p* < .001, *r* = .152), a higher economic success (1.60 vs. 1.45, U = 38694, *p* = .007, *r* = .106), and an overall higher perceived well-being (63.19% vs. 54.27%,U = 13106, *p* < .001, *r* = .150). Interestingly, those who used tactile maps for the last time at a younger age have the highest well-being scores (rho = 0.390, *p* = .005) and travel for more reasons (rho = 0.254, *p* = .026). Finally, those who are better at reading tactile maps have better cognitive mapping skills (rho = 0.376, *p* < .001), travel for more reasons (rho = 0.361, *p* < .001), and tend to be more confident traveling in unfamiliar areas (rho = 0.194, *p* = .064). Figure 3 illustrates a summary of these impacts.

### Specialized mobility services and tactile map usage

Since tactile maps are often used in specialized mobility training, such as O&M services, the impact of such services and their interaction with the usage of tactile maps were also investigated. To do so, we evaluated the effects of mobility services, tactile map usage, and both together, with the effect of neither as control. Of the 510 PVI who answered survey 2, 21 had only tactile map experience, 130 only received specialized mobility training, 57 benefited from both, and 302 received neither. Significant differences in all sets of variables except confidence in outdoor and indoor environments were detected (more details in supplementary file 2, Sect. 4): participants with experience in both mobility services and tactile maps often demonstrated superior mobility and well-being outcomes compared to those with only one type of experience and/or none.

Indeed, having access to both conferred advantages over either one in terms of the frequency of assistance needed during travel (compared to tactile map only: 2.28 vs. 2.83, z=-3.06, *p* = .003, *r*=-.213; compared to mobility services only: 2.28 vs. 2.52, z=-2.20, *p* = .036, *r*=-.102). Essentially, access to both was advantageous over specialized mobility services alone, but equivalent to tactile map experience alone in regards to improving cognitive mapping scores (compared to mobility services only: 57.46% vs. 45.62%, z = 3.13, *p* = .003, *r* = .166), confidence navigating unfamiliar environments (compared to mobility services only: 3.30 vs. 2.62, z = 3.13, *p* = .002, *r* = .155), and perceived environmental accessibility (compared to mobility services only: 2.86 vs. 1.89, z = 6.132, *p* < .001, *r* = .276). Furthermore, access to both showed some aspects in which it was advantageous over tactile map experience, but not over mobility services. These aspects included the number of reasons why participants travel (compared to tactile maps only: 3.98 vs. 2.83, z = 2.85, *p* = .013, *r* = .18) and their well-being scores (compared to tactile maps only: 70.53 vs. 45.00, z = 4.45, *p* < .001, *r* = .280).

Finally, in some areas, having access to both did not procure any advantages over either one. These included the frequency of travel (independent and accompanied), education level, and economic success. Surprisingly, tactile map experience alone was related to more frequent travels in unfamiliar areas compared to both combined (2.91 vs. 2.19, z = 2.787, *p* < .001, *r* = .199) and to mobility services only (2.91 vs. 2.25, z = 2.950, *p* < .001, *r* = .194). These findings suggest a cumulative advantage when both tactile maps and mobility services are given together, while each service offers distinct benefits.

**Fig. 4:**
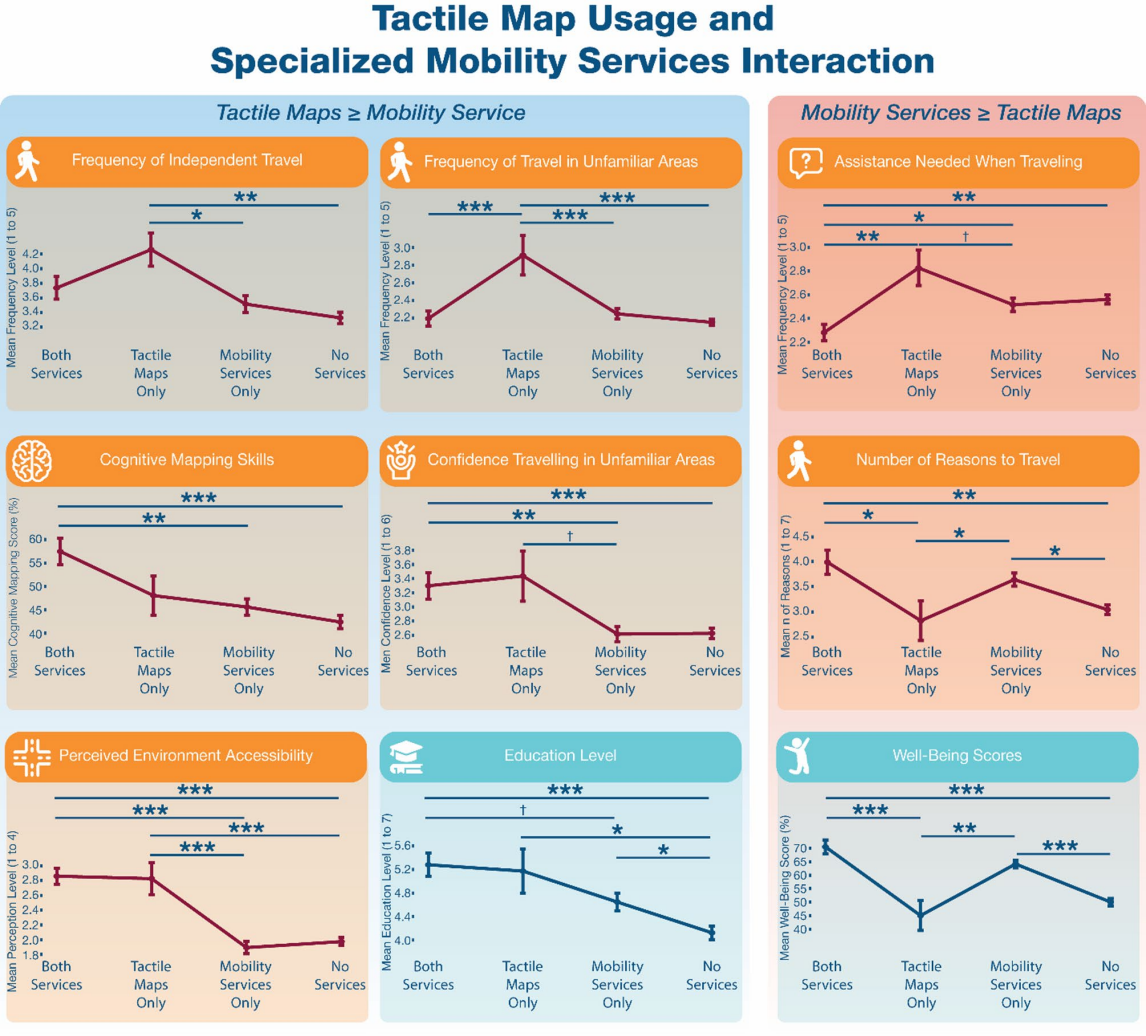
Interaction between tactile map usage and mobility services. Line plots display the means and standard error on the means (SEM) for nine aspects of mobility and well-being for which the Kruskal Wallis tests detected significant differences. The plots show the differences between those who received both services (tactile maps and mobility services), those who had access to only one, and those who received none are highlighted using the results of the post-hoc Dunn tests (see supplementary file 2). Mean ratings are on ordinal scales (i.e., 1 to 5, where each number represents a categorical, discrete level) and intermediate values are displayed for illustrative purposes (more information on the ordinal scales in Supplementary files 1 and 2). The well-being score (0-100%) reflects the quality, or level, of engagement in activities, personal connections, orientation capacities, and life space, while the cognitive mapping skills score (0-100%) reflects participants’ ability and confidence to learn the layout of new environments. Icons from Freepik (https://www.freepik.com). †*p* < .10 (non-significant trend); *p < .05; **p < .01; ***p < .001.

### Impacts of tactile maps according to the degree of functional vision

All participants whom did not identify as blind (*n* = 382), were categorized as having low vision (*n* = 382). Interestingly, blind and low vision participants were as likely as each other to have had experience with tactile maps (56/370 vs. 73/382, X^2^(1, *n* = 752) = 2.089, *p* = .148).

The 56 blind tactile map users, when compared to those with no experience, tend to be older (41.20y vs. 36.45y, U = 6894, *p* = .015, *r* = .134), to have had their VI diagnosed earlier (6.24y vs. 21.28y, U = 6011, *p* < .001, *r*=-.284) and to have become blind earlier in life (11.00y vs. 17.93y, U = 10772, *p* < .001, *r*=-.196). These participants travel independently more often (3.84 vs. 3.16, U = 6368, *p* = .001, *r* = .177), particularly more in familiar areas (3.70 vs. 3.29, U = 7124, *p* = .026, *r* = 124), travel for more reasons (3.82 vs. 3.00, U = 6561, *p* = .003, *r* = .124), perceive their environment as more accessible (2.79 vs. 1.80, U = 4338, *p* < .001, *r* = .334), and require help less often (2.31 vs. 2.72, U = 6544, *p* < .001, *r*=-.258). Along the same lines, blind tactile map users have better cognitive mapping skills (54.02% vs. 37.23%, U = 5326, *p* < .001, *r* = .245), a higher level of education (5.32 vs. 4.26, U = 6011, *p* < .001, *r* = .203), more economic success (1.57 vs. 1.39, U = 7340, *p* = .021, *r* = .120), and overall better well-being (66.67% vs. 51.09%, U = 2751, *p* < .001, *r* = .251). As it was the case with the general sample, those with earlier tactile map experience travel for more reasons (rho=-0.387, *p* = .023) and tended to have better well-being scores (rho=-0.388, *p* = .051, n.s. trend). Finally, those with progressive vision loss were less likely to have experience with tactile maps (29/261 vs. 27/109, X^2^(1,*n* = 370) = 11.169, *p* < .001).

As for the 73 tactile map users with low vision, they were also younger at the diagnosis of their visual condition (12.66y vs. 17.27y, U = 11272.5, *p* = .048, *r*=-.137). While they were not found to be more independent in their travels, they travel in unfamiliar areas more often (2.47 vs. 2.16, U = 9574, *p* = .048, *r* = .131) and find their environment more accessible (2.81 vs. 2.26, U = 7508, *p* < .001, *r* = .239). In contrast, they were also found to travel accompanied more often (3.03 vs. 2.54, U = 8205, *p* < .001, *r* = .197) and tended to require help more often (2.56 vs. 2.33, U = 3103, *p* = .082, n.s. trend, *r*=-.019). This result may be explained by the possibility that low-vision tactile map users have lower functional vision than the other low-vision participants. However, there were no such differences (2.73 vs. 2.81, U = 11997.5, *p* = .363, *r*=-.047); this may be because the nuances in residual vision could not be properly assessed from online self-reporting.

### Onset of visual impairments; the case of early- and late-onset blindness

**Fig. 5:**
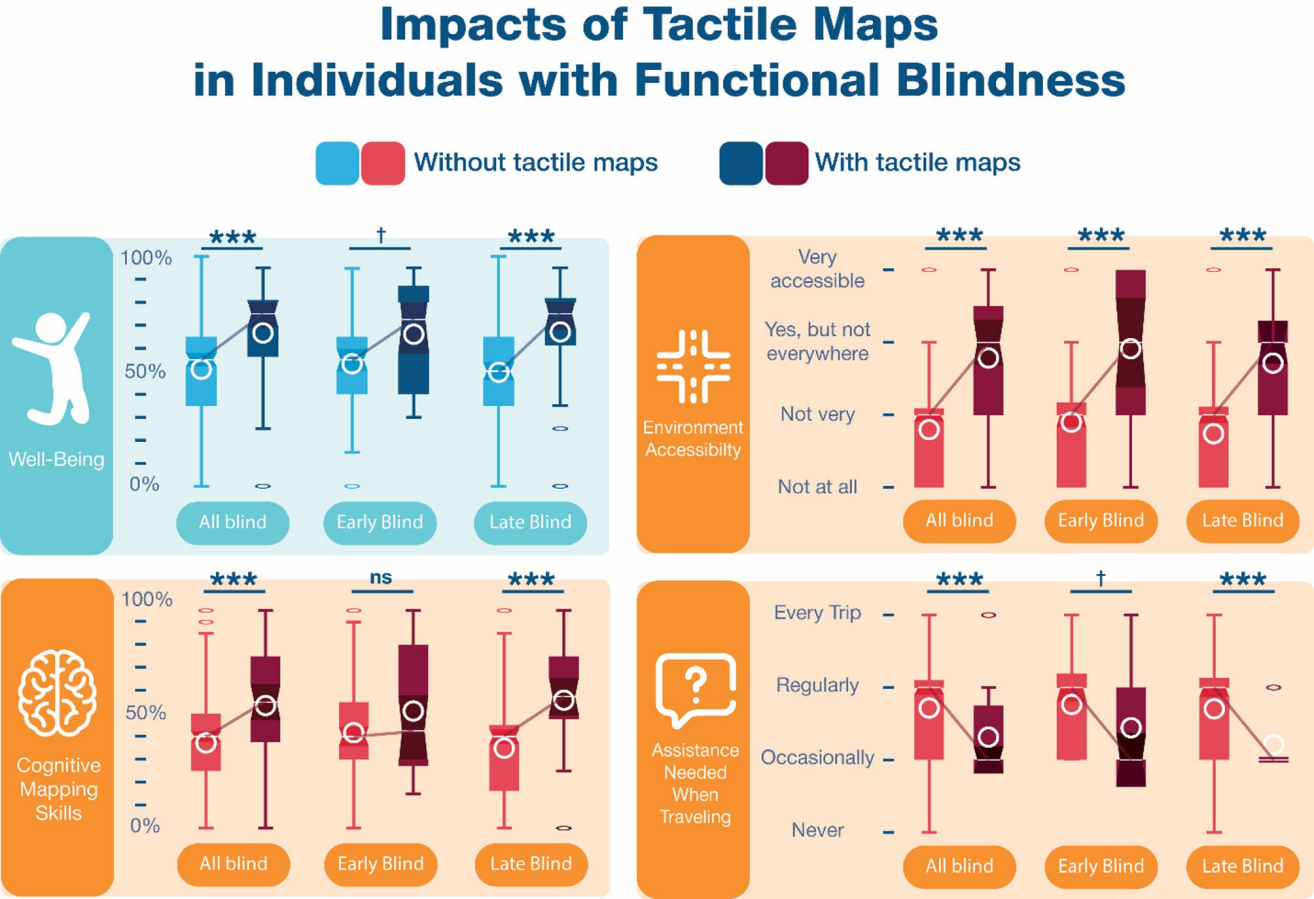
Impacts of tactile maps in people with functional blindness, including those with early- and late-onset blindness. Whisker plots display the differences between tactile map users (darker shades of blue and red) and the rest of the respondents (lighter shades of blue and red), including data medians (white bars) and means (white circles), notches illustrate the confidence interval around the median. Blue graphs refer to variables related to well-being and red graphs, to variables related to mobility and independence. The well-being score (0-100%) reflects the quality, or level, of engagement in activities, personal connections, orientation capacities, and life space, while the cognitive mapping skills score (0-100%) reflects participants’ ability and confidence to learn the layout of new environments. Icons from Freepik (https://www.freepik.com). *N.s.*, nonsignificant; †*p* < .10 (non-significant trend); *p < .05; **p < .01; ***p < .001.

Consistent to what was found in all participants who identified as blind, both EB and LB tactile map users had their visual impairment diagnosed earlier in life (EB: 1.71y vs. 2.73y, U = 1286.5, *p* = .004, *r*=-.323; LB: 10.06 vs. 19.32, U = 3606, *p* < .001, *r*=-.264), and EB tactile map users also experienced a significantly earlier onset of their blindness (0.77y vs. 2.38y, U = 1722, *p* < .001, *r*=-.361), Furthermore, EB were more likely to have prior experience with tactile maps than LB (26/115 vs. 28/223, X^2^(1,*n* = 338) = 5.712, *p* = .017). Figure 5 shows the significant effects of tactile maps in all blind participants, including EB and LB participants separately.

On average, EB tactile map users were first introduced to tactile maps at 10.96 years old (age range =^5,23^; see Fig. 4); in other words, 10.19 years after blindness onset. Their profiles paralleled those in the general sample as they find their environment more accessible (2.92 vs. 1.90, U = 565.5, *p* < .001, *r* = .386), and are more confident in unfamiliar areas (3.42 vs. 2.47, U = 710, *p* = .010, *r* = .298). The analyses also revealed non-significant trends: EB tactile map users generally tend to travel for more reasons (3.81 vs. 2.94, U = 790.5, *p* = .058, n.s. trend, *r* = .233), to require help less often (2.44 vs. 2.76, U = 794, *p* = .089, n.s. trend, *r*=-.232), and to have better well-being scores (U = 403.5, *p* = .089, n.s. trend, *r* = .239. Furthermore, when tactile maps are introduced earlier after diagnosis, EB tactile map users travel in unfamiliar areas more often (rho=-0.515, *p* = .049).

On average, LB tactile map users were first introduced to tactile maps at 22.75 years old (age range =^5,52^; see Fig. 5) or, in other words, 2.25 years after blindness onset. They travel independently more often (4.03 vs. 3.07, U = 1636.5, *p* < .001, *r* = .237), travel for more reasons (3.96 vs. 3.02, U = 1996, *p* = .033, *r* = .157), perceive their environment as more accessible (2.71 vs. 1.74, U = 1337, *p* < .001, *r* = .312), need help less often (2.21 vs. 2.70, U = 2064, *p* < .001, *r*=-.279), and have better confidence travelling in unfamiliar areas (3.39 vs. 2.36, U = 1609.5, *p* < .001, *r* = .257). Tactile maps in LB also were related to better cognitive mapping skills (55.89% vs. 34.92%, U = 1304.5, *p* < .001, *r* = .300), well-being (67.11% vs. 49.57%, U = 753.5, *p* < .001, *r* = .258), level of education (5.86 vs. 3.88, U = 1170.5, *p* < .001, *r* = .338), and tend to be related to a higher economic success (1.57 vs. 1.35, U = 2193, *p* = .063, n.s. trend, *r* = .134). Finally, LB with earlier exposure to tactile maps travel for more reasons (rho=-0.521, *p* = .037).

### Why are tactile maps not common?

As for the 623 respondents who never had experience with tactile maps, 621 provided the reasons why they did not. A multinomial test revealed that some reasons were more likely than others (X^2^(3, *n* = 621) = 206.7, *p* < .001). The most likely reason was that respondents did not know about tactile maps (*n* = 272, 43.8%), then that the level of their residual vision allowed them to function without needing tactile maps (*n* = 172, 27.7%), and that tactile maps were not available in their region (*n* = 160, 25.8%). Finally, “other reasons” were more unlikely (*n* = 18, 2.9%), but included various answers such as (1) non-specific reasons (they never used tactile maps; *n* = 12); (2) they think tactile maps are not practical (i.e., not useful, difficult to use, stressful, not transportable; *n* = 3) that they can manage with other tools (i.e., Google Maps; *n* = 2); and 4) that they fear equipment theft (*n* = 1).

## Discussion

The present study investigated the prevalence and global impacts of tactile map usage for PVI, with a specific focus on well-being. To do so, responses from the surveys were used to (1) paint the profiles of numerous PVI users around the world, including factors such as education, economic success, frequency of independent travel, perceived independence, and well-being outcomes; and (2) evaluate the link between those factors and the use of tactile maps.

### Prevalence and impacts of tactile maps

According to the results of this study, PVI, including individuals with low vision and blindness, can attain a functional level of independence and even exhibit varying levels of spatial abilities, which are influenced by different environmental, social, and personal factors, including the use of tactile maps. However, despite the known benefits of tactile maps on orientation abilities, this study reveals that their use is less prevalent than previously suspected. Indeed, only 17% of respondents had experience with these tools, which increased to 30% in those who received specialized mobility training. While this statistic varied according to regions and countries, it was explained mainly by the fact that participants’ knowledge of tactile maps was limited, which may, in turn, be caused by a lack of availability in many regions addressed in this survey. Nonetheless, the present study reveals the real impact that tactile maps can have in the daily lives of PVI worldwide, while other work has described their effects in controlled settings and tasks^16–20,23,44,45^.

The present results confirms that the usefulness of tactile maps is not limited to people with complete blindness but extend to those with varying levels of residual vision^46–48^. This includes improving cognitive mapping skills of PVI beyond laboratory settings, rendering the environment more accessible for PVI and, even, positively influencing independence, confidence, and well-being measures (e.g., engagement in activities, personal connections, orientation capacities, life space and self-determination)^43^. However, the impact on well-being was mostly due to the added support of specialized mobility training. This suggests that, considering the specialized needs of PVI for navigation, tactile maps should be fabricated and taught by those with specialized training, such as O&M specialists. This also highlights that specialized mobility services are essential for PVI to safely and efficiently navigate their environment and learn new routes. The subgroup analyses in the blind population also revealed that the tactile map effect on well-being and cognitive mapping skills was most significant for those with late onset blindness, consistent with the fact that prior visual experience facilitates map reading and spatial cognition^31–34^.

Nonetheless, while the combination of mobility services and tactile map usage provided the best outcomes, tactile maps seemed to have a broader impact than mobility services on participants’ confidence and spatial cognition skills. This emphasizes that tactile maps are, or should be, a crucial element within specialized mobility services, and that orientation training with tactile maps can be as crucial for independence as safe mobility training^31^. Giving access to such tools can be one of the most beneficial actions to help PVI fully develop spatial and problem-solving skills. Indeed, tactile map training can assist in planning navigation in unfamiliar environments, thus “visiting” these environments mentally prior to navigation in the real environments, raising confidence and increasing the frequency at which they visit these new environments (see Figs. 3 and 4). Consequently, tactile maps can support PVI in becoming independent, universal travelers and reaching social success, particularly educational success (see Fig. 3).

Consistently, the survey revealed the importance of early exposure to tactile maps for PVI and its benefits on independence and well-being, suggesting that tactile maps are tools that are not only useful for training cognitive mapping skills, but also to develop the understanding of spatial concepts in younger PVI^20,49–52^. These gains in spatial cognition can then be generalizable to new areas (i.e., new neighborhoods) and remain relevant in the long term. Indeed, in the case of EB individuals, the average age (around 10.9 years) at first tactile map introduction corresponded to the period they developed basic spatial skills and learned to be more independent^51,52^. Furthermore, the results also confirm that tactile graphics and maps of varying levels of complexity can be given to even younger participants (e.g., as early as five years old in the context of the present study) to train basic spatial concepts and abilities^48^, abilities that may also include the very spatial cognition skills believed to be requisites for using tactile maps^31^.

Finally, tactile maps, despite their rare use cases in the daily lives of PVI, emerged as relevant tools for the whole duration of their life and rehabilitation process. Indeed, using tactile maps in more concrete situations (i.e., when learning to walk around independently in a new area) leads to a better understanding of the environment and to better mobility measures and independence. However, a surprising outcome of this study was that those with progressive visual loss were less likely to have tactile map experience. Therefore, rehabilitation professionals should aim to introduce these tools before visual functions are too significantly reduced, which would capitalize on the remaining level of vision to facilitate the comprehension of tactile cartography via multisensory learning^46–48,53^.

Tactile maps are therefore revealed to be at their most useful when (1) receiving mobility training; (2) developing spatial cognition and orientation concepts; or (3) modifying travel needs (i.e., learning how to get to a new destination, learning the layout of a new environment such as a neighborhood, school, workplace, or grocery store). Table 1 presents, as advice to practitioners, general times and goals of tactile map training depending on the age and visual condition of PVI.

**Table 1:** Adapting tactile map training to the age and type of VI.

Type of VI	Tactile map intervention
Early-onset VI	Children (5 to 10 years old): simple tactile graphics and maps can support the development of basic spatial concepts and orientation skills (spatial awareness to different landmarks).
	Teenagers (11 to 19 years old): tactile graphics and maps can support the development of more advanced spatial concepts and orientation skills when teenagers first learn to become more independent in their travel, such as gaining more precise spatial knowledge from their environment (i.e., general layout, spatial relationships and landmarks)
	Adults (20 years old and older): tactile maps can help learn routes and gain more spatial knowledge about their environment.
Late-onset VI	As early as possible after diagnosis, introduction to tactile maps can help them with more specific training, like route travel, or gaining more spatial knowledge of their environment.
Progressive vision loss	Tactile maps can help PVI prepare and develop compensatory skills like tactile sensitivity and learning different rules of tactile maps making while they still have visual support.
Low vision	Tactile maps can provide a multisensory experience, complementing visual information and verbal descriptions when residual vision is limited.

### Improving access to tactile maps

However, most PVI still do not benefit from tactile maps. Therefore, providers of specialized mobility services, such as O&M specialists or teachers, should aim to give tactile map training more often to improve their clients’ or students’ confidence, independence, and general well-being. Similarly, PVI would benefit from enhanced interactions with tactile maps outside mobility services. However, this might not be possible due to the lack of available tactile maps. This is likely due to the complexity of tactile map production. Indeed, traditional tactile maps are often manually crafted by O&M or tactile graphic specialists, which is labor-intensive and time-consuming^54,55^. They also require significant data filtration and simplification (i.e., carefully selecting symbols and layers of information) to avoid clutter and maintain usability^31,46,56^. Furthermore, tactile maps must often be tailored to specific locations or purposes, a customization need that adds another layer of complexity^46^. Finally, tactile maps are typically produced using specialized technologies, which, combined with the manual nature of their production, contributes to high costs and lack of availability in institutions dealing with resource constraints^31,56,57^.

New research, therefore, investigates new ways and techniques to produce tactile maps more efficiently, for example, through automatic production either via open data (i.e., OpenStreetMap) or AI computer vision^57–61^, or through updatable digital tactile maps that can be interacted with via smartphone and tablet technology^60,62–64^. Some work also uses technologies such as 3D printing to improve the user experience and even the cognitive mapping process^32,65–70^. This research is vital as it would allow more PVI to benefit from tactile maps more quickly and at a lower cost. Further research will also need to define how these different types of tactile map technologies compare in terms of their impact on cognitive mapping skills and independent travel and what specific features contribute most significantly to their effectiveness.

Finally, the present study also investigated the impact of having access to publicly displayed tactile maps, generally in the form of city maps^65,71^ or floor plans^26,32,72^. Responses from the survey confirm that these tactile maps are not common or not accessible enough to provide an additional benefit for PVI independence, especially so for those with complete blindness or for those who could not explore their surroundings before their VI onset. Therefore, better universal accessibility guidelines^73^ are necessary to place such tactile maps in a more standardized way. For this, interactive audio-tactile maps could be considered as they allow PVI users to explore spatial information and enhance their ability to form cognitive maps through a multisensory experience^26,45,49,53,63,71,74–76^, while still being useful for other members of the society^53^.

### Limitations

The present study provides valuable insights from a large sample of participants and was conducted in four languages with the support of specialized associations to increase the study’s scope (e.g., avoiding Western bias) and help individuals with less technological competency. However, it is possible that the sample does not represent the views of those with lower technology competencies or those less connected to online services, while tactile map users may live in areas where visual disabilities are more considered in services and environmental design. Furthermore, as an online survey, no tests could be performed to confirm respondents’ level of vision or if they understood all questions and provided the most exact answers for their situation. Human assistance may also be a potential source of bias but was included to permit participants with low technological proficiencies to participate in our study. Lastly, due to the diversity in respondents’ profiles, regions, and life habits, this study cannot confirm or pinpoint the exact causes directly impacting mobility measures, well-being, and cognitive mapping abilities. Therefore, future studies will be warranted to confirm the present findings with direct and multifactorial testing with participants. Nonetheless, this study offers valuable context on how tactile map usage is related to various positive outcomes in the PVI population and can inform future investigators, rehabilitation professionals, and teachers aiming to provide the best spatial training to PVI worldwide.

## Conclusion

The present study is the first international survey to gather over 500 responses from six different continents and to explore the impact of tactile maps on the daily lives of people with visual impairments, with a particular focus on independent travel and well-being. This survey demonstrates that tactile maps are valuable tools for developing cognitive mapping skills and fostering a durable, generalizable understanding of diverse environments. It also highlights that tactile map interventions can enhance the subsequent development of spatial skills and promote lifelong navigational independence. Consequently, early exposure to tactile maps may be crucial during rehabilitation, especially at a young age—to aid in concept development—or before vision loss becomes too severe. This is especially pertinent as orientation continues to be a significant challenge PVI faces while new technologies are being tested to address the limited availability of tactile maps.

## Methods

### Inclusion criteria and ethical considerations

All data was gathered through two online surveys, published internationally on different social media channels and shared through specialized associations for people with visual impairments. Prior to responding to the actual survey questions, participants received information about the research and its purpose via an information sheet and were informed that responding to the survey would be considered as their given informed consent to participate in the study. Following this protocol, informed consent was obtained from all participants who answered the survey. To be retained as participants, respondents had to be at least 18 years old and self-identify as having a visual impairment. The study was conducted in accordance with the declaration of Helsinki. The ethics approval was obtained from the Comité d’éthique à la recherche Clinique (CERC) of the Université de Montréal (# 2023–5070).

### Survey description

Data was collected using two online surveys administered through LimeSurvey^77^ between December 2023 and September 2024. The survey was published in English, French, Spanish, and Mandarin; all versions were verified by native speakers with experience in vision rehabilitation. The survey was divided into two parts, and participants received the link to the second survey after having completed the first one. The full English version of the 2 surveys is available in Supplementary file S1.

Survey 1 was divided into 4 sections. Section 1 comprised 18 to 21 questions about sociodemographic factors such as age, gender, area of living, visual condition, age at diagnosis, degree of functional vision, and use of mobility aids. Section 2 comprised 19 to 27 questions about traveling habits, such as mobility aids, how frequently they travel outside the home (independently vs. accompanied, or in familiar vs. unfamiliar areas), and their confidence level when traveling. Section 3 comprised 7 questions and served as a questionnaire to score the cognitive mapping skills (on 100%) according to their confidence in various situations. Finally, Sect. 4 comprised 4 to 25 questions about participants’ experience with tactile and visual maps. This part of the survey collected data such as the first time they were introduced to those maps, how often they use them, in what context, for what purpose, and how good they are at reading them.

Survey 2 was divided into 4 sections. Section 1 comprised 2 to 17 questions about the reception of specialized mobility training such as O&M services, how often they need them, and what tools and/or strategies they learned. Section 2 comprised 10 to 15 questions about the various orientation strategies they use when travelling and how frequently they need assistance during their travels. Section 3 comprised 6 questions, in which participants were given scenarios about wayfinding in indoor and outdoor environments, explaining their strategies and rating their confidence in both types of environments. Finally, Sect. 4 was a questionnaire based on the OMO tool part B^43,78^, allowing the team to get measures of participants’ general well-being, based on engagement in activities, personal connections, orientation abilities, life space and self-determination.

### Data filtering

Results from the survey were exported to Microsoft Excel, where incomplete submissions and duplicates could be removed, with an expected attrition rate (or drop-out rate)^42^ of around 50% for both surveys, estimated according to the web-based nature of the survey^79^, its duration (over 30 min according to prior testing with PVI), and factors related to the added complexity of visual impairments and other disabilities such as platform accessibility, software or assistive technology issues, technological problems during participation, web navigation skills, and memory load when (re)reading questions and related multiple choices^80,81^. As expected, 685 of 1437 submissions were removed (47.67% attrition rate; including incomplete submission and duplicates) from survey 1, and 261 of 845 from survey 2 (30.88% attrition rate). This resulted in a total of 752 complete entries in the first survey and 584 complete entries in the second. Entries corresponding to the same individual were then matched together according to email and/or IP addresses. Following this methodology, 510 respondents completed both surveys, corresponding to a 32.18% drop-out rate from survey 1 to survey 2; while 74 entries in survey 2 could not be matched to entries in survey 1 using this matching method.

### Statistical analysis

From the participants’ answers, the main variables were extracted for analysis. First, participants were categorized based on whether they had prior experience with tactile maps and specialized mobility services. Then, their sociodemographic profiles, tactile map usage, mobility habits (including perceived independence), and well-being were assessed using specific sets of variables (see Table 2). Among these variables, two scores (in percentage) defined participants’ (1) cognitive mapping abilities, assessed using an original questionnaire in survey 1, Sect. 4 (6 questions); and (2) level of well-being, as measured by the OMO tool, Part B^43^ in survey 2, Sect. 4 (5 questions). These scores were calculated with participants’ answers to multiple questions (each on an ordinal scale, summed together) following these equations:

$$\:\text{C}\text{o}\text{g}\text{n}\text{i}\text{t}\text{i}\text{v}\text{e}\:\text{m}\text{a}\text{p}\text{p}\text{i}\text{n}\text{g}\:\text{s}\text{c}\text{o}\text{r}\text{e}=\left(\frac{{\sum\:}_{n=1}^{6}{Q}_{n}}{20}\right)\times\:100\%$$

$$\:\text{O}\text{M}\text{O}\:\text{s}\text{c}\text{o}\text{r}\text{e}=\left(\frac{{\sum\:}_{n=1}^{5}{Q}_{n}}{20}\right)\times\:100\%$$

As a result, the cognitive mapping score served as a measure of participants’ ability and confidence to learn the layout of new environments, while the OMO score, as a measure of well-being that considers their level of engagement in activities, the quality of their personal connections, their orientation capacities, the extent of their life space, and their level of self-determination. The data were then analyzed using non-parametric statistical tests in SPSS version 29, JASP^82^ and Python with the goal of evaluating the link between tactile map usage and sociodemographic factors, mobility & independence measures, and well-being.

**Table 2:** Variables used in the analysis, their data type, and corresponding survey questions (classified according to survey and section, see Supplementary file 1). S1, survey 1; S2, survey 2; Q, question.

Category	Variable	Data type	# Question
Socio demographics	Age	Continuous	S1, Q1.1
	Gender	Categorical	S1, Q1.2
	Country of residence	Categorical	S1, Q1.3
	Living area type	Categorical	S1, Q1.11
	Cause of VI	Categorical	S1, Q1.5
	If VI is or was gradual (progressive visual loss)	Binary	S1, Q1.7
	Age at VI diagnosis	Continuous	S1, Q1.6
	Utility of vision	Ordinal (4 levels)	S1, Q1.9
	Age at onset of blindness (if no usable vision)	Continuous	S1, Q1.10
Tactile map usage	Context of tactile map usage	Categorical	S1, Q 4.8
	Tactile map production method;	Categorical	S1, Q 4.6
	Types of maps (i.e., city map, floor plan);	Categorical	S1, Q 4.4
	Age at first tactile map	Continuous	S1, Q 4.3
	Time since last used	Ordinal (5 levels)	S1, Q 4.5
	Capacity to read tactile maps	Ordinal (5 levels)	S1, Q 4.11
	Goal of tactile map usage	Categorical	S1, Q 4.9–10
Mobility & independence	How frequently they travel independently	Ordinal (5 levels)	S1, Q2.1
	How frequently they travel accompanied	Ordinal (5 levels)	S1, Q2.2
	For how many reasons do they travel	Discrete (0 to 7)	S1, Q2.3
	How accessible they perceive their environment	Ordinal (4 levels)	S1, Q2.4
	How frequently they travel in familiar areas	Ordinal (5 levels)	S1, Q 2.5
	How frequently they travel in unfamiliar areas	Ordinal (5 levels)	S1, Q 2.6
	Confidence when travelling in unfamiliar areas	Ordinal (6 levels)	S1, Q2.7
	Confidence when travelling in outdoor areas	Ordinal (10 levels)	S2, Q3.1
	Confidence when travelling in indoor areas	Ordinal (10 levels)	S2, Q3.2
	How frequently they require help during travels	Ordinal (5 levels)	S2, Q2.1
	Cognitive mapping ability score	Continuous	S1, Q3.1-6
Well-being	Highest level of education completed	Ordinal (7 levels)	S1, Q1.4
	Economic status	Ordinal (3 levels)	S1, Q1.12
	Well-being (OMO) score	Continuous	S2, Q4.1-5

First, to define the usage of tactile maps worldwide and their relationship to sociodemographic factors, various statistical tests were employed. As most variables were categorical, relationships were examined using Chi-square tests of independence. For questions regarding the context of tactile map usage, production methods, and types of maps used, participants could select multiple options. Consequently, Cochran’s Q tests were performed to assess whether all options were chosen with equal frequency or if specific options were more common. When significant differences were found, pairwise post-hoc Dunn’s multiple comparisons tests (with Bonferroni corrections) were conducted to identify which options differed significantly. Participants without prior experience with tactile maps were also asked to provide the reason for their non-use. A multinomial test was conducted to determine whether specific reasons were reported more frequently than others.

Then, the main analyses aimed to establish how tactile map usage was related to mobility, independence and well-being. These statistical analyses aimed to uncover potential associations rather than establish cause. Given the number of comparisons and variable types (continuous and ordinal), non-parametric tests were prioritized, and p-values were adjusted using the Benjamini-Hochberg False Discovery Rate (FDR) correction to account for multiple comparisons. These analyses included the non-parametric Mann-Whitney U test to compare the sociodemographic profiles, mobility, and well-being of tactile map users and non-users; and the Spearman’s rank correlation coefficient (Spearman’s rho) to assess correlations between age at first tactile map use (and this age relative to participants’ visual impairment diagnosis), time elapsed since last tactile map use, and participants’ capacity or efficiency in reading tactile maps with relevant mobility and well-being outcomes (frequency of independent travel, number of reasons to travel, frequency of travel in unfamiliar areas, confidence when travelling in unfamiliar areas, perceived environment accessibility, cognitive mapping score, and well-being score). These analyses were conducted not only on the general sample but also within subgroups of participants with functional blindness and low vision, including separate analyses for individuals with early-onset blindness (onset before age six) and those with late-onset blindness (onset after age six). The same analyses were also conducted to assess the impact of access to public tactile maps (a solution designed to enhance environmental accessibility for individuals with visual impairments) by comparing the mobility and well-being of those exposed to public tactile maps with those who were not.

Finally, to ensure that the impact of tactile maps was not confounded by access to specialized mobility services, Kruskal-Wallis (non-parametric equivalent to ANOVAs) tests were conducted to detect any significant differences between those who benefited from both tactile maps and mobility services, those who benefited from either one and those who had none of these services. These were conducted for the same sets of variables as the main analyses and FDR corrections were also applied to account for multiple comparisons. Then, when the Kruskal-Wallis were significant, post-hoc Dunn tests with FDR correction were conducted to locate which sub-groups of participants differed from each other. This analysis allowed to discriminate between the effects of tactile maps and those of specialized mobility services, as well as to investigate how their effects compounded.

## Electronic supplementary material

Below is the link to the electronic supplementary material.

Supplementary Material 1

Supplementary Material 2

## Data Availability

The data and all materials for the experiments reported here are available. Access to the data can be requested by contacting the corresponding author.
