# Supplementary material for: International prevalence of tactile map usage and its impact on navigational independence and well-being of people with visual impairments
Source: Sci Rep. 2025 Jul 26;15:27245. doi: 10.1038/s41598-025-08117-9 (PMC12297645; doi:10.1038/s41598-025-08117-9)
Supplement: Supplementary file 2 — Supplementary Material 2 [file 41598_2025_8117_MOESM2_ESM.docx]

**1. Variables tested**

**Demographic variables**

1. **Age:** age (in years) at participation
2. **Education:** Highest level completed (ordinal: 1=lowest, 7=highest)
3. **AgeDiagnosis:** age (in years) when VI was diagnosed
4. **EconomicClass:** economic status (ordinal: 1: lowest, 3: highest)
5. **UtilityVisionGroup:** how vision is useful for navigation (ordinal: 1=lowest, 4=highest)
6. **BlindnessONSET:** age (in years) when participants became functionally blind

**Mobility and well-being variables**

1. **FreqTravelindependan:** How frequently they travel independently (ordinal: 1=lowest, 5=highest)
2. **FreqTravelAccompany:** How frequently they travel accompanied (ordinal: 1=lowest, 5=highest)
3. **nTravelReasons:** For how many reasons do they travel (discrete from 1 to 7 reasons)
4. **AccessWalkInfrastruc:** How accessible they perceive their environment (ordinal: 1=lowest, 4=highest)
5. **ScoreCognitiveMapSkills:** Cognitive mapping ability score (in %)
6. **FreqTravelFamiliar:** How frequently they travel in familiar areas (ordinal: 1=lowest, 5=highest)
7. **FreqTravelUnfamiliar:** How frequently they travel in unfamiliar areas (ordinal: 1=lowest, 5=highest)
8. **ConfidenceUnfamiliar:** Confidence when travelling in unfamiliar areas (ordinal: 1=lowest, 6=highest)
9. **FreqHelp:** How frequently they require help during travels (ordinal: 1=lowest, 5=highest)
10. **OutdoorConf:** Confidence when travelling in outdoor areas (ordinal: 1=lowest, 10=highest)
11. **IndoorConf:** Confidence when travelling in indoor areas (ordinal: 1=lowest, 10=highest)
12. **OMOscore:** Well-being (OMO) score (in %)
13. **Education:** Highest level completed (ordinal: 1=lowest, 7=highest)
14. **EconomicClass:** economic status (ordinal: 1: lowest, 3: highest)

**Variables used for correlation analysis**

1. **AgeFirstTactMap:** age (in years) when first introduced to tactile maps
2. **TactMapCapacity:** self-rated capacity to read tactile maps (ordinal: 1=lowest, 5=highest)
3. **TactMapLastUse:** Time since last used (ordinal: 1=most recent, 5=least recent)
4. **TactMapVdiagnosis:** time between first introduction and VI diagnosis = (AgeFirstTactMap - AgeDiagnosis)

**2. Main analyses: Mann-Whitney tests to compare tactile map users to non-users**

**Table S1. Demographics in the general PVI sample**

| Variable | U statistic | p-value | Effect Size (r) | Mean Non-Users | Mean Users | FDR-corrected p-value | Significance |
| --- | --- | --- | --- | --- | --- | --- | --- |
| Age | 38684 | 0.504292 | 0.02435 | 36.28892 | 37.13953 | 0.504292 | n.s. |
| Education | 31161 | 3.21E-05 | 0.151617 | 4.399679 | 5.147287 | 9.64E-05 | *** |
| AgeDiagnosis | 43594 | 2.57E-07 | -0.19572 | 19.25407 | 9.780172 | 1.54E-06 | *** |
| EconomicClass | 34530.5 | 0.003728 | 0.105764 | 1.449438 | 1.604651 | 0.005591 | ** |
| UtilityVisionGroup | 38093.5 | 0.316657 | 0.036516 | 1.897271 | 1.976744 | 0.379989 | n.s. |
| BlindnessONSET | 10034 | 0.00032 | -0.19571 | 17.92958 | 11 | 0.000641 | *** |

**Table S2: Mobility and Well-Being measures in the general PVI sample**

| Variable | U statistic | p-value | Effect Size (r) | Mean Non-Users | Mean Users | FDR-corrected p-value | Significance |
| --- | --- | --- | --- | --- | --- | --- | --- |
| FreqTravelindependan | 35082.5 | 0.018201 | 0.086115 | 3.35634 | 3.674419 | 0.028312 | * |
| FreqTravelAccompany | 31792 | 6.38E-05 | 0.145803 | 2.611557 | 2.992248 | 0.000179 | *** |
| nTravelReasons | 35707 | 0.04257 | 0.07395 | 3.101124 | 3.488372 | 0.059598 | n.s. |
| AccessWalkInfrastruc | 22901 | 9.49E-16 | 0.292944 | 2.025682 | 2.79845 | 1.33E-14 | *** |
| ScoreCognitiveMapSkills | 30827 | 2.96E-05 | 0.152302 | 44.11717 | 53.52713 | 0.00015 | *** |
| FreqTravelFamiliar | 38694 | 0.485622 | 0.025427 | 3.414125 | 3.488372 | 0.485622 | n.s. |
| FreqTravelUnfamiliar | 35012.5 | 0.002876 | 0.108693 | 2.194222 | 2.395349 | 0.005753 | ** |
| ConfidenceUnfamiliar | 31684 | 5.69E-05 | 0.146782 | 2.691814 | 3.248062 | 0.000179 | *** |
| FreqHelp | 19077 | 0.083261 | -0.0767 | 2.546512 | 2.4375 | 0.097138 | n.s. |
| OutdoorConf | 19287.5 | 0.081426 | -0.07716 | 7.868744 | 6.25 | 0.097138 | n.s. |
| IndoorConf | 18066.5 | 0.469701 | -0.03201 | 6.410465 | 6.3625 | 0.485622 | n.s. |
| OMOscore | 13106 | 0.000701 | 0.150078 | 54.26744 | 63.1875 | 0.001635 | ** |
| Education | 31161 | 3.21E-05 | 0.151617 | 4.399679 | 5.147287 | 0.00015 | *** |
| EconomicClass | 34530.5 | 0.003728 | 0.105764 | 1.449438 | 1.604651 | 0.006523 | ** |

**Table S3. Demographics in respondents who are functionally blind**

| Variable | U statistic | p-value | Effect Size (r) | Mean Non-Users | Mean Users | FDR-corrected p-value | Significance |
| --- | --- | --- | --- | --- | --- | --- | --- |
| Age | 6894 | 0.010033 | 0.133852 | 36.45223 | 41.19643 | 0.015049 | * |
| Education | 6011 | 9.36E-05 | 0.203102 | 4.261146 | 5.321429 | 0.000281 | *** |
| AgeDiagnosis | 10772 | 1.89E-07 | -0.28379 | 21.28421 | 6.240385 | 1.13E-06 | *** |
| EconomicClass | 7340 | 0.021037 | 0.119952 | 1.388535 | 1.571429 | 0.025244 | * |
| UtilityVisionGroup | 8792 | 1 | 0 | 1 | 1 | 1 | n.s. |
| BlindnessONSET | 10034 | 0.00032 | -0.19571 | 17.92958 | 11 | 0.000641 | *** |

**Table S4: Mobility and Well-Being measures in respondents who are functionally blind**

| Variable | U statistic | p-value | Effect Size (r) | Mean Non-Users | Mean Users | FDR-corrected p-value | Significance |
| --- | --- | --- | --- | --- | --- | --- | --- |
| FreqTravelindependan | 6368 | 0.00066 | 0.17706 | 3.16242 | 3.839286 | 0.001319 | ** |
| FreqTravelAccompany | 7625 | 0.08769 | 0.08878 | 2.681529 | 2.946429 | 0.102305 | n.s. |
| nTravelReasons | 6561 | 0.001973 | 0.160863 | 3.003185 | 3.821429 | 0.003453 | ** |
| AccessWalkInfrastruc | 4338 | 1.39E-10 | 0.333622 | 1.796178 | 2.785714 | 1.94E-09 | *** |
| ScoreCognitiveMapSkills | 5326 | 2.41E-06 | 0.245166 | 37.2293 | 54.01786 | 1.12E-05 | *** |
| FreqTravelFamiliar | 7124.5 | 0.016822 | 0.12428 | 3.286624 | 3.696429 | 0.026168 | * |
| FreqTravelUnfamiliar | 7879 | 0.104549 | 0.084386 | 2.232484 | 2.303571 | 0.112591 | n.s. |
| ConfidenceUnfamiliar | 5174.5 | 8.63E-08 | 0.278315 | 2.404459 | 3.392857 | 6.04E-07 | *** |
| FreqHelp | 6544 | 1.52E-05 | -0.25758 | 2.716049 | 2.307692 | 5.32E-05 | *** |
| OutdoorConf | 5676 | 0.044557 | -0.11962 | 6.884774 | 6.128205 | 0.056708 | n.s. |
| IndoorConf | 5251 | 0.27169 | -0.06546 | 6.600823 | 6.282051 | 0.27169 | n.s. |
| OMOscore | 2751 | 2.52E-05 | 0.250897 | 51.09053 | 66.66667 | 7.05E-05 | *** |
| Education | 6011 | 9.36E-05 | 0.203102 | 4.261146 | 5.321429 | 0.000218 | *** |
| EconomicClass | 7340 | 0.021037 | 0.119952 | 1.388535 | 1.571429 | 0.029452 | * |

**Table S5. Demographics in respondents who have low vision**

| Variable | U statistic | p-value | Effect Size (r) | Mean Non-Users | Mean Users | FDR-corrected p-value | Significance |
| --- | --- | --- | --- | --- | --- | --- | --- |
| Age | 12371.5 | 0.197724 | -0.0659 | 36.12298 | 34.0274 | 0.247155 | n.s. |
| Education | 9679 | 0.051143 | 0.099785 | 4.540453 | 5.013699 | 0.127857 | n.s. |
| AgeDiagnosis | 11272.5 | 0.009665 | -0.13714 | 17.2726 | 12.65625 | 0.048325 | * |
| EconomicClass | 10076 | 0.102884 | 0.083451 | 1.511327 | 1.630137 | 0.171474 | n.s. |
| UtilityVisionGroup | 11997.5 | 0.363043 | -0.04654 | 2.809061 | 2.726027 | 0.363043 | n.s. |

**Table S6: Mobility and Well-Being measures who have low vision**

| Variable | U statistic | p-value | Effect Size (r) | Mean Non-Users | Mean Users | FDR-corrected p-value | Significance |
| --- | --- | --- | --- | --- | --- | --- | --- |
| FreqTravelindependan | 11395.5 | 0.885686 | -0.00736 | 3.553398 | 3.547945 | 0.953816 | n.s. |
| FreqTravelAccompany | 8205.5 | 0.00012 | 0.196781 | 2.540453 | 3.027397 | 0.00084 | *** |
| nTravelReasons | 11255.5 | 0.978523 | 0.001377 | 3.200647 | 3.232877 | 0.978523 | n.s. |
| AccessWalkInfrastruc | 7508.5 | 3.14E-06 | 0.238505 | 2.2589 | 2.808219 | 4.39E-05 | *** |
| ScoreCognitiveMapSkills | 10689.5 | 0.486898 | 0.035572 | 51.1165 | 53.15068 | 0.852071 | n.s. |
| FreqTravelFamiliar | 12430.5 | 0.155128 | -0.07274 | 3.543689 | 3.328767 | 0.310257 | n.s. |
| FreqTravelUnfamiliar | 9574.5 | 0.010224 | 0.131398 | 2.15534 | 2.465753 | 0.047714 | * |
| ConfidenceUnfamiliar | 10872 | 0.616639 | 0.025614 | 2.983819 | 3.136986 | 0.860717 | n.s. |
| FreqHelp | 3103 | 0.023297 | 0.150237 | 2.326203 | 2.560976 | 0.08154 | n.s. |
| OutdoorConf | 3944.5 | 0.770862 | -0.01929 | 9.14738 | 6.365854 | 0.899339 | n.s. |
| IndoorConf | 3674.5 | 0.676278 | 0.027653 | 6.163102 | 6.439024 | 0.860717 | n.s. |
| OMOscore | 3641 | 0.61491 | 0.033317 | 58.39572 | 59.87805 | 0.860717 | n.s. |
| Education | 9679 | 0.051143 | 0.099785 | 4.540453 | 5.013699 | 0.1432 | n.s. |
| EconomicClass | 10076 | 0.102884 | 0.083451 | 1.511327 | 1.630137 | 0.240064 | n.s. |

**Table S7. Demographics in respondents who have early blindness**

| Variable | U statistic | p-value | Effect Size (r) | Mean Non-Users | Mean Users | FDR-corrected p-value | Significance |
| --- | --- | --- | --- | --- | --- | --- | --- |
| Age | 851 | 0.040763 | 0.190785 | 30.94382 | 37.19231 | 0.081525 | n.s. |
| Education | 1202 | 0.756736 | -0.02889 | 4.910112 | 4.807692 | 0.908083 | n.s. |
| AgeDiagnosis | 1286.5 | 0.00131 | -0.32301 | 2.733333 | 1.708333 | 0.003929 | ** |
| EconomicClass | 1009 | 0.254294 | 0.106304 | 1.426966 | 1.576923 | 0.381441 | n.s. |
| UtilityVisionGroup | 1157 | 1 | 0 | 1 | 1 | 1 | n.s. |
| BlindnessONSET | 1722 | 0.00011 | -0.36062 | 2.382022 | 0.769231 | 0.000661 | *** |

**Table S8: Mobility and Well-Being measures who have early blindness**

| Variable | U statistic | p-value | Effect Size (r) | Mean Non-Users | Mean Users | FDR-corrected p-value | Significance |
| --- | --- | --- | --- | --- | --- | --- | --- |
| FreqTravelindependan | 1074 | 0.563485 | 0.053868 | 3.494382 | 3.692308 | 0.657399 | n.s. |
| FreqTravelAccompany | 1005 | 0.271742 | 0.102488 | 2.696629 | 2.923077 | 0.42271 | n.s. |
| nTravelReasons | 790.5 | 0.012355 | 0.233297 | 2.94382 | 3.807692 | 0.057659 | n.s. |
| AccessWalkInfrastruc | 565.5 | 3.53E-05 | 0.385695 | 1.898876 | 2.923077 | 0.000495 | *** |
| ScoreCognitiveMapSkills | 952.5 | 0.170996 | 0.127661 | 41.68539 | 51.15385 | 0.341992 | n.s. |
| FreqTravelFamiliar | 1106.5 | 0.724772 | 0.032832 | 3.539326 | 3.653846 | 0.756736 | n.s. |
| FreqTravelUnfamiliar | 1078.5 | 0.525983 | 0.059135 | 2.348315 | 2.384615 | 0.657399 | n.s. |
| ConfidenceUnfamiliar | 710 | 0.001402 | 0.297859 | 2.47191 | 3.423077 | 0.009816 | ** |
| FreqHelp | 794 | 0.031609 | -0.23177 | 2.764706 | 2.444444 | 0.088505 | n.s. |
| OutdoorConf | 693 | 0.384016 | -0.09387 | 6.867647 | 6.111111 | 0.537623 | n.s. |
| IndoorConf | 768 | 0.089389 | -0.18317 | 6.911765 | 5.888889 | 0.208575 | n.s. |
| OMOscore | 403.5 | 0.026577 | 0.239138 | 53.23529 | 66.11111 | 0.088505 | n.s. |
| Education | 1202 | 0.756736 | -0.02889 | 4.910112 | 4.807692 | 0.756736 | n.s. |
| EconomicClass | 1009 | 0.254294 | 0.106304 | 1.426966 | 1.576923 | 0.42271 | n.s. |

**Table S9. Demographics in respondents who have late blindness**

| Variable | U statistic | p-value | Effect Size (r) | Mean Non-Users | Mean Users | FDR-corrected p-value | Significance |
| --- | --- | --- | --- | --- | --- | --- | --- |
| Age | 2139 | 0.064273 | 0.123904 | 40.06667 | 45.07143 | 0.09641 | n.s. |
| Education | 1170.5 | 4.53E-07 | 0.337846 | 3.882051 | 5.857143 | 2.72E-06 | *** |
| AgeDiagnosis | 3606 | 0.000111 | -0.26356 | 19.32275 | 10.05769 | 0.000334 | *** |
| EconomicClass | 2193 | 0.04529 | 0.13406 | 1.353846 | 1.571429 | 0.09058 | n.s. |
| UtilityVisionGroup | 2730 | 1 | 0 | 1 | 1 | 1 | n.s. |
| BlindnessONSET | 3242 | 0.108788 | -0.10739 | 25.02564 | 20.5 | 0.130545 | n.s. |

**Table S10: Mobility and Well-Being measures who have late blindness**

| Variable | U statistic | p-value | Effect Size (r) | Mean Non-Users | Mean Users | FDR-corrected p-value | Significance |
| --- | --- | --- | --- | --- | --- | --- | --- |
| FreqTravelindependan | 1636.5 | 0.000391 | 0.237464 | 3.071795 | 4.035714 | 0.000912 | *** |
| FreqTravelAccompany | 2265.5 | 0.116505 | 0.105109 | 2.625641 | 3 | 0.14828 | n.s. |
| nTravelReasons | 1996 | 0.018865 | 0.157246 | 3.020513 | 3.964286 | 0.033014 | * |
| AccessWalkInfrastruc | 1337 | 3.06E-06 | 0.312487 | 1.74359 | 2.714286 | 2.15E-05 | *** |
| ScoreCognitiveMapSkills | 1304.5 | 7.43E-06 | 0.300071 | 34.92308 | 55.89286 | 3.47E-05 | *** |
| FreqTravelFamiliar | 2120 | 0.042753 | 0.135679 | 3.205128 | 3.678571 | 0.063406 | n.s. |
| FreqTravelUnfamiliar | 2448.5 | 0.24048 | 0.078603 | 2.2 | 2.25 | 0.258978 | n.s. |
| ConfidenceUnfamiliar | 1609.5 | 0.000122 | 0.257282 | 2.358974 | 3.392857 | 0.000427 | *** |
| FreqHelp | 2064.5 | 0.000285 | -0.27909 | 2.7 | 2.210526 | 0.000799 | *** |
| OutdoorConf | 1718 | 0.140531 | -0.11337 | 6.713333 | 6 | 0.163952 | n.s. |
| IndoorConf | 1396 | 0.885908 | 0.011037 | 6.266667 | 6.578947 | 0.885908 | n.s. |
| OMOscore | 753.5 | 0.000813 | 0.257565 | 49.56667 | 67.10526 | 0.001626 | ** |
| Education | 1170.5 | 4.53E-07 | 0.337846 | 3.882051 | 5.857143 | 6.35E-06 | *** |
| EconomicClass | 2193 | 0.04529 | 0.13406 | 1.353846 | 1.571429 | 0.063406 | n.s. |

**3. Main analyses: Spearman Rho correlations**

**Table S11: Correlation tests in the general sample**

| Variable 1 | Variable 2 | | | | Spearman Correlation | | | | Raw p-value | | | | Significance-uncorrected | | | | FDR-corrected p-value | | | | Significance-corrected | | | |  |  |  |
| --- | --- | --- | --- | --- | --- | --- | --- | --- | --- | --- | --- | --- | --- | --- | --- | --- | --- | --- | --- | --- | --- | --- | --- | --- | --- | --- | --- |
| AgeFirstTactMap | nTravelReasons | | | | -0.13912 | | | | 0.115861 | | | | n.s. | | | | 0.2732 | | | | n.s. | | | |  |  |  |
| AgeFirstTactMap | AccessWalkInfrastruc | | | | -0.03461 | | | | 0.696968 | | | | n.s. | | | | 0.81313 | | | | n.s. | | | |  |  |  |
| AgeFirstTactMap | ScoreCognitiveMapSkills | | | | -0.14522 | | | | 0.100584 | | | | n.s. | | | | 0.2732 | | | | n.s. | | | |  |  |  |
| AgeFirstTactMap | FreqTravelindependan | | | | -0.10456 | | | | 0.238282 | | | | n.s. | | | | 0.333595 | | | | n.s. | | | |  |  |  |
| AgeFirstTactMap | FreqTravelUnfamiliar | | | | -0.01276 | | | | 0.885881 | | | | n.s. | | | | 0.885881 | | | | n.s. | | | |  |  |  |
| AgeFirstTactMap | ConfidenceUnfamiliar | | | | -0.10467 | | | | 0.237785 | | | | n.s. | | | | 0.333595 | | | | n.s. | | | |  |  |  |
| AgeFirstTactMap | OMOscore | | | | -0.17661 | | | | 0.117086 | | | | n.s. | | | | 0.2732 | | | | n.s. | | | |  |  |  |
|  | | | | | | | | | | | | | | | | | | | | | | | | | |  |  |
| TactMapLastUse | | nTravelReasons | | | | 0.253805 | | | | 0.003704 | | | | ** | | | | 0.012966 | | | | * | | | |  |  |
| TactMapLastUse | | AccessWalkInfrastruc | | | | -0.01463 | | | | 0.869301 | | | | n.s. | | | | 0.869301 | | | | n.s. | | | |  |  |
| TactMapLastUse | | ScoreCognitiveMapSkills | | | | 0.111727 | | | | 0.207463 | | | | n.s. | | | | 0.363061 | | | | n.s. | | | |  |  |
| TactMapLastUse | | FreqTravelindependan | | | | 0.084918 | | | | 0.338657 | | | | n.s. | | | | 0.3951 | | | | n.s. | | | |  |  |
| TactMapLastUse | | FreqTravelUnfamiliar | | | | -0.17309 | | | | 0.049805 | | | | * | | | | 0.116211 | | | | n.s. | | | |  |  |
| TactMapLastUse | | ConfidenceUnfamiliar | | | | 0.099713 | | | | 0.260883 | | | | n.s. | | | | 0.365236 | | | | n.s. | | | |  |  |
| TactMapLastUse | | OMOscore | | | | 0.389595 | | | | 0.000354 | | | | *** | | | | 0.00248 | | | | ** | | | |  |  |
|  | | | | | | | | | | | | | | | | | | | | | | | | | | |  |
| TactMapCapacity | | | nTravelReasons | | | | 0.360831 | | | | 2.66E-05 | | | | *** | | | | 9.31E-05 | | | | *** | | | |  |
| TactMapCapacity | | | AccessWalkInfrastruc | | | | 0.050986 | | | | 0.566085 | | | | n.s. | | | | 0.660432 | | | | n.s. | | | |  |
| TactMapCapacity | | | ScoreCognitiveMapSkills | | | | 0.376407 | | | | 1.10E-05 | | | | *** | | | | 7.71E-05 | | | | *** | | | |  |
| TactMapCapacity | | | FreqTravelindependan | | | | 0.071607 | | | | 0.420001 | | | | n.s. | | | | 0.588002 | | | | n.s. | | | |  |
| TactMapCapacity | | | FreqTravelUnfamiliar | | | | -0.01171 | | | | 0.895234 | | | | n.s. | | | | 0.895234 | | | | n.s. | | | |  |
| TactMapCapacity | | | ConfidenceUnfamiliar | | | | 0.194281 | | | | 0.027371 | | | | * | | | | 0.063865 | | | | n.s. | | | |  |
| TactMapCapacity | | | OMOscore | | | | 0.21105 | | | | 0.060216 | | | | n.s. | | | | 0.105379 | | | | n.s. | | | |  |
|  | | | | | | | | | | | | | | | | | | | | | | | | | | | |
| TactMapVdiagnosis | | | | nTravelReasons | | | | 0.148392 | | | | 0.093287 | | | | n.s. | | | | 0.480532 | | | | n.s. | | | |
| TactMapVdiagnosis | | | | AccessWalkInfrastruc | | | | -0.07175 | | | | 0.419075 | | | | n.s. | | | | 0.769477 | | | | n.s. | | | |
| TactMapVdiagnosis | | | | ScoreCognitiveMapSkills | | | | -0.0662 | | | | 0.456033 | | | | n.s. | | | | 0.769477 | | | | n.s. | | | |
| TactMapVdiagnosis | | | | FreqTravelindependan | | | | 0.021407 | | | | 0.809714 | | | | n.s. | | | | 0.944666 | | | | n.s. | | | |
| TactMapVdiagnosis | | | | FreqTravelUnfamiliar | | | | -0.13154 | | | | 0.137295 | | | | n.s. | | | | 0.480532 | | | | n.s. | | | |
| TactMapVdiagnosis | | | | ConfidenceUnfamiliar | | | | -0.0049 | | | | 0.956075 | | | | n.s. | | | | 0.956075 | | | | n.s. | | | |
| TactMapVdiagnosis | | | | OMOscore | | | | -0.06789 | | | | 0.549626 | | | | n.s. | | | | 0.769477 | | | | n.s. | | | |

**Table S12: Correlation tests in functionally blind respondents**

| Variable 1 | Variable 2 | | | | Spearman Correlation | | | | Raw p-value | | | | Significance-uncorrected | | | | FDR-corrected p-value | | | | Significance-corrected | | | |  |  |  |
| --- | --- | --- | --- | --- | --- | --- | --- | --- | --- | --- | --- | --- | --- | --- | --- | --- | --- | --- | --- | --- | --- | --- | --- | --- | --- | --- | --- |
| AgeFirstTactMap | nTravelReasons | | | | -0.38697 | | | | 0.003217 | | | | ** | | | | 0.02252 | | | | * | | | |  |  |  |
| AgeFirstTactMap | AccessWalkInfrastruc | | | | 0.003761 | | | | 0.97805 | | | | n.s. | | | | 0.97805 | | | | n.s. | | | |  |  |  |
| AgeFirstTactMap | ScoreCognitiveMapSkills | | | | -0.1852 | | | | 0.171778 | | | | n.s. | | | | 0.386863 | | | | n.s. | | | |  |  |  |
| AgeFirstTactMap | FreqTravelindependan | | | | -0.11635 | | | | 0.393122 | | | | n.s. | | | | 0.458642 | | | | n.s. | | | |  |  |  |
| AgeFirstTactMap | FreqTravelUnfamiliar | | | | -0.14801 | | | | 0.276331 | | | | n.s. | | | | 0.386863 | | | | n.s. | | | |  |  |  |
| AgeFirstTactMap | ConfidenceUnfamiliar | | | | -0.1502 | | | | 0.269206 | | | | n.s. | | | | 0.386863 | | | | n.s. | | | |  |  |  |
| AgeFirstTactMap | OMOscore | | | | -0.38821 | | | | 0.014601 | | | | * | | | | 0.051103 | | | | n.s. | | | |  |  |  |
|  | | | | | | | | | | | | | | | | | | | | | | | | | | |  |
| TactMapCapacity | | | nTravelReasons | | | | 0.288874 | | | | 0.030831 | | | | * | | | | 0.10791 | | | | n.s. | | | |  |
| TactMapCapacity | | | AccessWalkInfrastruc | | | | 0.232533 | | | | 0.084599 | | | | n.s. | | | | 0.197399 | | | | n.s. | | | |  |
| TactMapCapacity | | | ScoreCognitiveMapSkills | | | | 0.304042 | | | | 0.022718 | | | | * | | | | 0.10791 | | | | n.s. | | | |  |
| TactMapCapacity | | | FreqTravelindependan | | | | 0.029262 | | | | 0.830484 | | | | n.s. | | | | 0.947368 | | | | n.s. | | | |  |
| TactMapCapacity | | | FreqTravelUnfamiliar | | | | -0.00902 | | | | 0.947368 | | | | n.s. | | | | 0.947368 | | | | n.s. | | | |  |
| TactMapCapacity | | | ConfidenceUnfamiliar | | | | 0.011546 | | | | 0.932692 | | | | n.s. | | | | 0.947368 | | | | n.s. | | | |  |
| TactMapCapacity | | | OMOscore | | | | 0.153078 | | | | 0.352181 | | | | n.s. | | | | 0.616317 | | | | n.s. | | | |  |
|  | | | | | | | | | | | | | | | | | | | | | | | | | |  |  |
| TactMapLastUse | | nTravelReasons | | | | 0.127497 | | | | 0.349061 | | | | n.s. | | | | 0.610856 | | | | n.s. | | | |  |  |
| TactMapLastUse | | AccessWalkInfrastruc | | | | -0.01751 | | | | 0.898063 | | | | n.s. | | | | 0.932717 | | | | n.s. | | | |  |  |
| TactMapLastUse | | ScoreCognitiveMapSkills | | | | 0.130837 | | | | 0.336476 | | | | n.s. | | | | 0.610856 | | | | n.s. | | | |  |  |
| TactMapLastUse | | FreqTravelindependan | | | | -0.01154 | | | | 0.932717 | | | | n.s. | | | | 0.932717 | | | | n.s. | | | |  |  |
| TactMapLastUse | | FreqTravelUnfamiliar | | | | -0.16822 | | | | 0.21522 | | | | n.s. | | | | 0.610856 | | | | n.s. | | | |  |  |
| TactMapLastUse | | ConfidenceUnfamiliar | | | | 0.027266 | | | | 0.841892 | | | | n.s. | | | | 0.932717 | | | | n.s. | | | |  |  |
| TactMapLastUse | | OMOscore | | | | 0.342384 | | | | 0.032878 | | | | * | | | | 0.230144 | | | | n.s. | | | |  |  |
|  | | | | | | | | | | | | | | | | | | | | | | | | | | | |
| TactMapVdiagnosis | | | | nTravelReasons | | | | -0.16205 | | | | 0.232768 | | | | n.s. | | | | 0.543125 | | | | n.s. | | | |
| TactMapVdiagnosis | | | | AccessWalkInfrastruc | | | | 0.065889 | | | | 0.629471 | | | | n.s. | | | | 0.683709 | | | | n.s. | | | |
| TactMapVdiagnosis | | | | ScoreCognitiveMapSkills | | | | -0.19055 | | | | 0.159522 | | | | n.s. | | | | 0.543125 | | | | n.s. | | | |
| TactMapVdiagnosis | | | | FreqTravelindependan | | | | -0.07458 | | | | 0.584852 | | | | n.s. | | | | 0.683709 | | | | n.s. | | | |
| TactMapVdiagnosis | | | | FreqTravelUnfamiliar | | | | -0.1267 | | | | 0.352118 | | | | n.s. | | | | 0.616206 | | | | n.s. | | | |
| TactMapVdiagnosis | | | | ConfidenceUnfamiliar | | | | -0.05566 | | | | 0.683709 | | | | n.s. | | | | 0.683709 | | | | n.s. | | | |
| TactMapVdiagnosis | | | | OMOscore | | | | -0.22498 | | | | 0.168499 | | | | n.s. | | | | 0.543125 | | | | n.s. | | | |

**Table S13: Correlation tests in low vision participants**

| Variable 1 | Variable 2 | | | | Spearman Correlation | | | | Raw p-value | | | | Significance-uncorrected | | | | FDR-corrected p-value | | | | Significance-corrected | | |  |  |
| --- | --- | --- | --- | --- | --- | --- | --- | --- | --- | --- | --- | --- | --- | --- | --- | --- | --- | --- | --- | --- | --- | --- | --- | --- | --- |
| AgeFirstTactMap | nTravelReasons | | | | 0.086429 | | | | 0.467186 | | | | n.s. | | | | 0.816006 | | | | n.s. | | |  |  |
| AgeFirstTactMap | AccessWalkInfrastruc | | | | -0.08159 | | | | 0.492589 | | | | n.s. | | | | 0.816006 | | | | n.s. | | |  |  |
| AgeFirstTactMap | ScoreCognitiveMapSkills | | | | -0.12754 | | | | 0.282239 | | | | n.s. | | | | 0.816006 | | | | n.s. | | |  |  |
| AgeFirstTactMap | FreqTravelindependan | | | | -0.09081 | | | | 0.444812 | | | | n.s. | | | | 0.816006 | | | | n.s. | | |  |  |
| AgeFirstTactMap | FreqTravelUnfamiliar | | | | 0.034206 | | | | 0.773884 | | | | n.s. | | | | 0.816006 | | | | n.s. | | |  |  |
| AgeFirstTactMap | ConfidenceUnfamiliar | | | | -0.05774 | | | | 0.627546 | | | | n.s. | | | | 0.816006 | | | | n.s. | | |  |  |
| AgeFirstTactMap | OMOscore | | | | 0.037486 | | | | 0.816006 | | | | n.s. | | | | 0.816006 | | | | n.s. | | |  |  |
|  | | | | | | | | | | | | | | | | | | | | | | | | | |
| TactMapCapacity | | | nTravelReasons | | | | 0.403034 | | | | 0.000407 | | | | *** | | | | 0.001426 | | | ** | | | |
| TactMapCapacity | | | AccessWalkInfrastruc | | | | -0.10714 | | | | 0.36696 | | | | n.s. | | | | 0.513744 | | | n.s. | | | |
| TactMapCapacity | | | ScoreCognitiveMapSkills | | | | 0.431576 | | | | 0.000138 | | | | *** | | | | 0.000964 | | | *** | | | |
| TactMapCapacity | | | FreqTravelindependan | | | | 0.055104 | | | | 0.643337 | | | | n.s. | | | | 0.75056 | | | n.s. | | | |
| TactMapCapacity | | | FreqTravelUnfamiliar | | | | -0.01673 | | | | 0.888256 | | | | n.s. | | | | 0.888256 | | | n.s. | | | |
| TactMapCapacity | | | ConfidenceUnfamiliar | | | | 0.321405 | | | | 0.00556 | | | | ** | | | | 0.012973 | | | * | | | |
| TactMapCapacity | | | OMOscore | | | | 0.252825 | | | | 0.110749 | | | | n.s. | | | | 0.19381 | | | n.s. | | | |
|  | | | | | | | | | | | | | | | | | | | | | | | | |  |
| TactMapLastUse | | nTravelReasons | | | | 0.248755 | | | | 0.033824 | | | | * | | | | 0.118385 | | | | n.s. | | |  |
| TactMapLastUse | | AccessWalkInfrastruc | | | | -0.07075 | | | | 0.551952 | | | | n.s. | | | | 0.643944 | | | | n.s. | | |  |
| TactMapLastUse | | ScoreCognitiveMapSkills | | | | 0.054825 | | | | 0.645026 | | | | n.s. | | | | 0.645026 | | | | n.s. | | |  |
| TactMapLastUse | | FreqTravelindependan | | | | 0.0997 | | | | 0.401342 | | | | n.s. | | | | 0.643944 | | | | n.s. | | |  |
| TactMapLastUse | | FreqTravelUnfamiliar | | | | -0.16099 | | | | 0.173631 | | | | n.s. | | | | 0.405138 | | | | n.s. | | |  |
| TactMapLastUse | | ConfidenceUnfamiliar | | | | 0.086841 | | | | 0.465055 | | | | n.s. | | | | 0.643944 | | | | n.s. | | |  |
| TactMapLastUse | | OMOscore | | | | 0.337273 | | | | 0.031049 | | | | * | | | | 0.118385 | | | | n.s. | | |  |
|  | | | | | | | | | | | | | | | | | | | | | | |  |  |  |
| TactMapVdiagnosis | | | | nTravelReasons | | | | 0.297128 | | | | 0.010687 | | | | * | | | | 0.074808 | | n.s. |  |  |  |
| TactMapVdiagnosis | | | | AccessWalkInfrastruc | | | | -0.15395 | | | | 0.193472 | | | | n.s. | | | | 0.540009 | | n.s. |  |  |  |
| TactMapVdiagnosis | | | | ScoreCognitiveMapSkills | | | | 0.004594 | | | | 0.969227 | | | | n.s. | | | | 0.998725 | | n.s. |  |  |  |
| TactMapVdiagnosis | | | | FreqTravelindependan | | | | 0.051712 | | | | 0.66393 | | | | n.s. | | | | 0.929502 | | n.s. |  |  |  |
| TactMapVdiagnosis | | | | FreqTravelUnfamiliar | | | | -0.1418 | | | | 0.231433 | | | | n.s. | | | | 0.540009 | | n.s. |  |  |  |
| TactMapVdiagnosis | | | | ConfidenceUnfamiliar | | | | -0.00019 | | | | 0.998725 | | | | n.s. | | | | 0.998725 | | n.s. |  |  |  |
| TactMapVdiagnosis | | | | OMOscore | | | | 0.079753 | | | | 0.620128 | | | | n.s. | | | | 0.929502 | | n.s. |  |  |  |

**Table S14: Correlation tests in early blind participants**

| Variable 1 | Variable 2 | | | | Spearman Correlation | | | | Raw p-value | | | | Significance-uncorrected | | | | FDR-corrected p-value | | | | Significance-corrected | | | |  |  |  |
| --- | --- | --- | --- | --- | --- | --- | --- | --- | --- | --- | --- | --- | --- | --- | --- | --- | --- | --- | --- | --- | --- | --- | --- | --- | --- | --- | --- |
| AgeFirstTactMap | nTravelReasons | | | | -0.37381 | | | | 0.059949 | | | | n.s. | | | | 0.13988 | | | | n.s. | | | |  |  |  |
| AgeFirstTactMap | AccessWalkInfrastruc | | | | 0.400065 | | | | 0.042859 | | | | * | | | | 0.13988 | | | | n.s. | | | |  |  |  |
| AgeFirstTactMap | ScoreCognitiveMapSkills | | | | -0.13592 | | | | 0.507922 | | | | n.s. | | | | 0.592576 | | | | n.s. | | | |  |  |  |
| AgeFirstTactMap | FreqTravelindependan | | | | -0.13612 | | | | 0.507311 | | | | n.s. | | | | 0.592576 | | | | n.s. | | | |  |  |  |
| AgeFirstTactMap | FreqTravelUnfamiliar | | | | -0.25783 | | | | 0.203501 | | | | n.s. | | | | 0.356128 | | | | n.s. | | | |  |  |  |
| AgeFirstTactMap | ConfidenceUnfamiliar | | | | 0.0269 | | | | 0.896216 | | | | n.s. | | | | 0.896216 | | | | n.s. | | | |  |  |  |
| AgeFirstTactMap | OMOscore | | | | -0.54275 | | | | 0.019944 | | | | * | | | | 0.139608 | | | | n.s. | | | |  |  |  |
|  | | | | | | | | | | | | | | | | | | | | | | | | | | |  |
| TactMapCapacity | | | nTravelReasons | | | | 0.300444 | | | | 0.135875 | | | | n.s. | | | | 0.451541 | | | | n.s. | | | |  |
| TactMapCapacity | | | AccessWalkInfrastruc | | | | 0.296748 | | | | 0.140993 | | | | n.s. | | | | 0.451541 | | | | n.s. | | | |  |
| TactMapCapacity | | | ScoreCognitiveMapSkills | | | | 0.263422 | | | | 0.193518 | | | | n.s. | | | | 0.451541 | | | | n.s. | | | |  |
| TactMapCapacity | | | FreqTravelindependan | | | | 0.104831 | | | | 0.610293 | | | | n.s. | | | | 0.748789 | | | | n.s. | | | |  |
| TactMapCapacity | | | FreqTravelUnfamiliar | | | | 0.06598 | | | | 0.748789 | | | | n.s. | | | | 0.748789 | | | | n.s. | | | |  |
| TactMapCapacity | | | ConfidenceUnfamiliar | | | | -0.06957 | | | | 0.735605 | | | | n.s. | | | | 0.748789 | | | | n.s. | | | |  |
| TactMapCapacity | | | OMOscore | | | | 0.196012 | | | | 0.435676 | | | | n.s. | | | | 0.748789 | | | | n.s. | | | |  |
|  | | | | | | | | | | | | | | | | | | | | | | | | | |  |  |
| TactMapLastUse | | nTravelReasons | | | | 0.279816 | | | | 0.16622 | | | | n.s. | | | | 0.646355 | | | | n.s. | | | |  |  |
| TactMapLastUse | | AccessWalkInfrastruc | | | | 0.077808 | | | | 0.705574 | | | | n.s. | | | | 0.818229 | | | | n.s. | | | |  |  |
| TactMapLastUse | | ScoreCognitiveMapSkills | | | | 0.080556 | | | | 0.695656 | | | | n.s. | | | | 0.818229 | | | | n.s. | | | |  |  |
| TactMapLastUse | | FreqTravelindependan | | | | 0.183579 | | | | 0.369346 | | | | n.s. | | | | 0.646355 | | | | n.s. | | | |  |  |
| TactMapLastUse | | FreqTravelUnfamiliar | | | | -0.24986 | | | | 0.218314 | | | | n.s. | | | | 0.646355 | | | | n.s. | | | |  |  |
| TactMapLastUse | | ConfidenceUnfamiliar | | | | -0.04738 | | | | 0.818229 | | | | n.s. | | | | 0.818229 | | | | n.s. | | | |  |  |
| TactMapLastUse | | OMOscore | | | | 0.257771 | | | | 0.301743 | | | | n.s. | | | | 0.646355 | | | | n.s. | | | |  |  |
|  | | | | | | | | | | | | | | | | | | | | | | | | | | | |
| TactMapVdiagnosis | | | | nTravelReasons | | | | -0.35092 | | | | 0.078792 | | | | n.s. | | | | 0.275773 | | | | n.s. | | | |
| TactMapVdiagnosis | | | | AccessWalkInfrastruc | | | | 0.236072 | | | | 0.245626 | | | | n.s. | | | | 0.308598 | | | | n.s. | | | |
| TactMapVdiagnosis | | | | ScoreCognitiveMapSkills | | | | -0.27254 | | | | 0.177972 | | | | n.s. | | | | 0.308598 | | | | n.s. | | | |
| TactMapVdiagnosis | | | | FreqTravelindependan | | | | -0.22712 | | | | 0.264513 | | | | n.s. | | | | 0.308598 | | | | n.s. | | | |
| TactMapVdiagnosis | | | | FreqTravelUnfamiliar | | | | -0.51534 | | | | 0.007053 | | | | ** | | | | 0.049373 | | | | * | | | |
| TactMapVdiagnosis | | | | ConfidenceUnfamiliar | | | | 0.00623 | | | | 0.975904 | | | | n.s. | | | | 0.975904 | | | | n.s. | | | |
| TactMapVdiagnosis | | | | OMOscore | | | | -0.31648 | | | | 0.200724 | | | | n.s. | | | | 0.308598 | | | | n.s. | | | |

**Table S15: Correlation tests in late blind participants**

| Variable 1 | Variable 2 | | | | Spearman Correlation | | | | Raw p-value | | | | Significance-uncorrected | | | | FDR-corrected p-value | | | | Significance-corrected | | | |  |  |  |
| --- | --- | --- | --- | --- | --- | --- | --- | --- | --- | --- | --- | --- | --- | --- | --- | --- | --- | --- | --- | --- | --- | --- | --- | --- | --- | --- | --- |
| AgeFirstTactMap | nTravelReasons | | | | -0.51197 | | | | 0.005352 | | | | ** | | | | 0.037466 | | | | * | | | |  |  |  |
| AgeFirstTactMap | AccessWalkInfrastruc | | | | -0.04073 | | | | 0.836975 | | | | n.s. | | | | 0.976471 | | | | n.s. | | | |  |  |  |
| AgeFirstTactMap | ScoreCognitiveMapSkills | | | | -0.39405 | | | | 0.038003 | | | | * | | | | 0.125035 | | | | n.s. | | | |  |  |  |
| AgeFirstTactMap | FreqTravelindependan | | | | -0.23226 | | | | 0.23432 | | | | n.s. | | | | 0.328048 | | | | n.s. | | | |  |  |  |
| AgeFirstTactMap | FreqTravelUnfamiliar | | | | 0.004036 | | | | 0.983738 | | | | n.s. | | | | 0.983738 | | | | n.s. | | | |  |  |  |
| AgeFirstTactMap | ConfidenceUnfamiliar | | | | -0.28215 | | | | 0.145767 | | | | n.s. | | | | 0.255092 | | | | n.s. | | | |  |  |  |
| AgeFirstTactMap | OMOscore | | | | -0.44937 | | | | 0.053586 | | | | n.s. | | | | 0.125035 | | | | n.s. | | | |  |  |  |
|  | | | | | | | | | | | | | | | | | | | | | | | | | | |  |
| TactMapCapacity | | | nTravelReasons | | | | 0.224613 | | | | 0.250511 | | | | n.s. | | | | 0.670644 | | | | n.s. | | | |  |
| TactMapCapacity | | | AccessWalkInfrastruc | | | | 0.122183 | | | | 0.535663 | | | | n.s. | | | | 0.670644 | | | | n.s. | | | |  |
| TactMapCapacity | | | ScoreCognitiveMapSkills | | | | 0.426391 | | | | 0.023659 | | | | * | | | | 0.165611 | | | | n.s. | | | |  |
| TactMapCapacity | | | FreqTravelindependan | | | | -0.14245 | | | | 0.469607 | | | | n.s. | | | | 0.670644 | | | | n.s. | | | |  |
| TactMapCapacity | | | FreqTravelUnfamiliar | | | | -0.16774 | | | | 0.393546 | | | | n.s. | | | | 0.670644 | | | | n.s. | | | |  |
| TactMapCapacity | | | ConfidenceUnfamiliar | | | | 0.110729 | | | | 0.574838 | | | | n.s. | | | | 0.670644 | | | | n.s. | | | |  |
| TactMapCapacity | | | OMOscore | | | | 0.09581 | | | | 0.69641 | | | | n.s. | | | | 0.69641 | | | | n.s. | | | |  |
|  | | | | | | | | | | | | | | | | | | | | | | | | | |  |  |
| TactMapLastUse | | nTravelReasons | | | | 0.083628 | | | | 0.672233 | | | | n.s. | | | | 0.967462 | | | | n.s. | | | |  |  |
| TactMapLastUse | | AccessWalkInfrastruc | | | | 0.008077 | | | | 0.967462 | | | | n.s. | | | | 0.967462 | | | | n.s. | | | |  |  |
| TactMapLastUse | | ScoreCognitiveMapSkills | | | | 0.059124 | | | | 0.765052 | | | | n.s. | | | | 0.967462 | | | | n.s. | | | |  |  |
| TactMapLastUse | | FreqTravelindependan | | | | -0.14479 | | | | 0.462262 | | | | n.s. | | | | 0.967462 | | | | n.s. | | | |  |  |
| TactMapLastUse | | FreqTravelUnfamiliar | | | | -0.03436 | | | | 0.86221 | | | | n.s. | | | | 0.967462 | | | | n.s. | | | |  |  |
| TactMapLastUse | | ConfidenceUnfamiliar | | | | 0.051305 | | | | 0.795422 | | | | n.s. | | | | 0.967462 | | | | n.s. | | | |  |  |
| TactMapLastUse | | OMOscore | | | | 0.501106 | | | | 0.028846 | | | | * | | | | 0.20192 | | | | n.s. | | | |  |  |
|  | | | | | | | | | | | | | | | | | | | | | | | | | | | |
| TactMapVdiagnosis | | | | nTravelReasons | | | | -0.1205 | | | | 0.541326 | | | | n.s. | | | | 0.81164 | | | | n.s. | | | |
| TactMapVdiagnosis | | | | AccessWalkInfrastruc | | | | -0.07733 | | | | 0.695692 | | | | n.s. | | | | 0.81164 | | | | n.s. | | | |
| TactMapVdiagnosis | | | | ScoreCognitiveMapSkills | | | | -0.09261 | | | | 0.639261 | | | | n.s. | | | | 0.81164 | | | | n.s. | | | |
| TactMapVdiagnosis | | | | FreqTravelindependan | | | | -0.03792 | | | | 0.848091 | | | | n.s. | | | | 0.848091 | | | | n.s. | | | |
| TactMapVdiagnosis | | | | FreqTravelUnfamiliar | | | | 0.136494 | | | | 0.488573 | | | | n.s. | | | | 0.81164 | | | | n.s. | | | |
| TactMapVdiagnosis | | | | ConfidenceUnfamiliar | | | | -0.20763 | | | | 0.289048 | | | | n.s. | | | | 0.81164 | | | | n.s. | | | |
| TactMapVdiagnosis | | | | OMOscore | | | | -0.15722 | | | | 0.520354 | | | | n.s. | | | | 0.81164 | | | | n.s. | | | |

**4. Kruskal-Wallis tests to investigate the effects of tactile maps and mobility services**

**Table S16: Kruskal-Wallis results with FDR correction**

| Variable | Mean Both | Mean Only Tactile Map | Mean Only Mobility  Service | Mean None | H statistic | p-value | FDR-corrected p-value | Significance |
| --- | --- | --- | --- | --- | --- | --- | --- | --- |
| FreqTravelindependan | 3.73684 | 4.26087 | 3.51538 | 3.32666 | 13.9858 | 0.00292 | 0.003722 | ** |
| FreqTravelAccompany | 2.78947 | 3.39130 | 2.57692 | 2.50333 | 14.7290 | 0.00206 | 0.00321 | ** |
| nTravelReasons | 3.98245 | 2.82608 | 3.63846 | 3.04333 | 24.3311 | 2.13E-05 | 5.96E-05 | *** |
| AccessWalkInfrastruc | 2.85964 | 2.82608 | 1.89230 | 1.97333 | 58.7376 | 1.09E-12 | 7.66E-12 | *** |
| ScoreCognitiveMapSkills | 57.4561 | 48.0434 | 45.6153 | 42.4166 | 20.6884 | 0.000122 | 0.000285 | *** |
| FreqTravelFamiliar | 3.70175 | 3.52173 | 3.62307 | 3.34 | 10.0021 | 0.018548 | 0.021639 | * |
| FreqTravelUnfamiliar | 2.19298 | 2.91304 | 2.24615 | 2.15 | 25.0768 | 1.49E-05 | 5.21E-05 | *** |
| ConfidenceUnfamiliar | 3.29824 | 3.43478 | 2.61538 | 2.62666 | 19.6354 | 0.000202 | 0.000404 | *** |
| FreqHelp | 2.28070 | 2.82608 | 2.51538 | 2.56 | 15.4481 | 0.001471 | 0.002574 | ** |
| OutdoorConf | 5.89473 | 7.13043 | 6.60430 | 8.41666 | 6.38436 | 0.094336 | 0.101593 | n.s. |
| IndoorConf | 6.01754 | 7.21739 | 6.54230 | 6.35333 | 2.97130 | 0.39607 | 0.39607 | n.s. |
| OMOscore | 70.5263 | 45 | 64.1538 | 49.9833 | 62.4063 | 1.80E-13 | 2.52E-12 | *** |
| Education | 5.28070 | 5.17391 | 4.64615 | 4.12333 | 25.1496 | 1.44E-05 | 5.21E-05 | *** |
| EconomicClass | 1.596491 | 1.347826 | 1.453846 | 1.336667 | 14.30265 | 0.002521 | 0.003529 | ** |

**Table S17: Pairwise post-hoc Dunn comparison tests**

| Variable | Group 1 | Group 2 | Z statistic | p-value | Effect Size (r) | FDR-corrected p-value | Significance |
| --- | --- | --- | --- | --- | --- | --- | --- |
| FreqTravelindependan | Both | Only Tactile Map | -1.52873 | 0.110048 | -0.12433 | 0.192584 | n.s. |
| FreqTravelindependan | Both | Only Mobility Service | 1.01554 | 0.288443 | 0.045884 | 0.410665 | n.s. |
| FreqTravelindependan | Both | None | 2.035338 | 0.033376 | 0.086462 | 0.073778 | n.s. |
| FreqTravelindependan | Only Tactile Map | Only Mobility Service | 2.382594 | 0.012756 | 0.15301 | 0.036143 | * |
| FreqTravelindependan | Only Tactile Map | None | 3.104649 | 0.001173 | 0.192391 | 0.00448 | ** |
| FreqTravelindependan | Only Mobility Service | None | 1.264317 | 0.186305 | 0.038013 | 0.289808 | n.s. |
|  | | | | | | | |
| FreqTravelAccompany | Both | Only Tactile Map | -1.08288 | 0.237781 | -0.1106 | 0.350414 | n.s. |
| FreqTravelAccompany | Both | Only Mobility Service | 1.777371 | 0.052662 | 0.082928 | 0.102874 | n.s. |
| FreqTravelAccompany | Both | None | 2.3752 | 0.009613 | 0.10114 | 0.02884 | * |
| FreqTravelAccompany | Only Tactile Map | Only Mobility Service | 2.43072 | 0.00805 | 0.156522 | 0.026008 | * |
| FreqTravelAccompany | Only Tactile Map | None | 2.82257 | 0.00209 | 0.171159 | 0.007632 | ** |
| FreqTravelAccompany | Only Mobility Service | None | 0.579411 | 0.527601 | 0.017654 | 0.624204 | n.s. |
|  | | | | | | | |
| nTravelReasons | Both | Only Tactile Map | 2.846335 | 0.003623 | 0.176964 | 0.012681 | * |
| nTravelReasons | Both | Only Mobility Service | 0.9939 | 0.309695 | 0.060796 | 0.433573 | n.s. |
| nTravelReasons | Both | None | 3.628512 | 0.000208 | 0.14693 | 0.00125 | ** |
| nTravelReasons | Only Tactile Map | Only Mobility Service | -2.41029 | 0.013757 | -0.15502 | 0.036143 | * |
| nTravelReasons | Only Tactile Map | None | -0.82658 | 0.398198 | -0.05775 | 0.499233 | n.s. |
| nTravelReasons | Only Mobility Service | None | 3.489343 | 0.000362 | 0.108949 | 0.002026 | ** |
|  | | | | | | | |
| AccessWalkInfrastruc | Both | Only Tactile Map | 0.384574 | 0.686463 | -0.00191 | 0.739268 | n.s. |
| AccessWalkInfrastruc | Both | Only Mobility Service | 6.131945 | 1.22E-10 | 0.276451 | 6.88E-09 | *** |
| AccessWalkInfrastruc | Both | None | 6.089737 | 1.64E-10 | 0.258684 | 6.88E-09 | *** |
| AccessWalkInfrastruc | Only Tactile Map | Only Mobility Service | 3.886291 | 4.52E-05 | 0.242977 | 0.000422 | *** |
| AccessWalkInfrastruc | Only Tactile Map | None | 3.627765 | 0.00014 | 0.226014 | 0.000907 | *** |
| AccessWalkInfrastruc | Only Mobility Service | None | -0.89723 | 0.346317 | -0.02903 | 0.476895 | n.s. |
|  | | | | | | | |
| ScoreCognitiveMapSkills | Both | Only Tactile Map | 1.517614 | 0.128087 | 0.118612 | 0.219578 | n.s. |
| ScoreCognitiveMapSkills | Both | Only Mobility Service | 3.344192 | 0.000799 | 0.166262 | 0.003355 | ** |
| ScoreCognitiveMapSkills | Both | None | 4.468768 | 7.44E-06 | 0.180263 | 9.54E-05 | *** |
| ScoreCognitiveMapSkills | Only Tactile Map | Only Mobility Service | 0.691237 | 0.488251 | 0.047157 | 0.585901 | n.s. |
| ScoreCognitiveMapSkills | Only Tactile Map | None | 1.251613 | 0.209488 | 0.079348 | 0.319945 | n.s. |
| ScoreCognitiveMapSkills | Only Mobility Service | None | 1.089824 | 0.274503 | 0.035641 | 0.397556 | n.s. |
|  | | | | | | | |
| FreqTravelFamiliar | Both | Only Tactile Map | 0.727744 | 0.439173 | 0.054539 | 0.542508 | n.s. |
| FreqTravelFamiliar | Both | Only Mobility Service | 0.541225 | 0.565074 | 0.023617 | 0.641436 | n.s. |
| FreqTravelFamiliar | Both | None | 2.294707 | 0.014717 | 0.09614 | 0.036359 | * |
| FreqTravelFamiliar | Only Tactile Map | Only Mobility Service | -0.41463 | 0.659391 | -0.02793 | 0.719336 | n.s. |
| FreqTravelFamiliar | Only Tactile Map | None | 0.701557 | 0.455816 | 0.04471 | 0.554906 | n.s. |
| FreqTravelFamiliar | Only Mobility Service | None | 2.338813 | 0.012913 | 0.070667 | 0.036143 | * |
|  | | | | | | | |
| FreqTravelUnfamiliar | Both | Only Tactile Map | -2.78653 | 0.00014 | -0.1987 | 0.000907 | *** |
| FreqTravelUnfamiliar | Both | Only Mobility Service | -0.13249 | 0.856343 | -0.00628 | 0.88806 | n.s. |
| FreqTravelUnfamiliar | Both | None | 0.651769 | 0.373174 | 0.027281 | 0.497565 | n.s. |
| FreqTravelUnfamiliar | Only Tactile Map | Only Mobility Service | 2.949927 | 5.56E-05 | 0.194147 | 0.000467 | *** |
| FreqTravelUnfamiliar | Only Tactile Map | None | 3.61676 | 7.74E-07 | 0.22558 | 1.30E-05 | *** |
| FreqTravelUnfamiliar | Only Mobility Service | None | 1.097314 | 0.133792 | 0.033359 | 0.22477 | n.s. |
|  | | | | | | | |
| ConfidenceUnfamiliar | Both | Only Tactile Map | 0.278844 | 0.765204 | -0.01907 | 0.803464 | n.s. |
| ConfidenceUnfamiliar | Both | Only Mobility Service | 3.283621 | 0.000437 | 0.154588 | 0.00226 | ** |
| ConfidenceUnfamiliar | Both | None | 3.617904 | 0.000107 | 0.152368 | 0.000814 | *** |
| ConfidenceUnfamiliar | Only Tactile Map | Only Mobility Service | 2.00147 | 0.03206 | 0.12709 | 0.073778 | n.s. |
| ConfidenceUnfamiliar | Only Tactile Map | None | 2.097741 | 0.024655 | 0.125362 | 0.059171 | n.s. |
| ConfidenceUnfamiliar | Only Mobility Service | None | 0.01063 | 0.990916 | 0.000769 | 0.990916 | n.s. |
|  | | | | | | | |
| FreqHelp | Both | Only Tactile Map | -3.06358 | 0.00062 | -0.2132 | 0.002743 | ** |
| FreqHelp | Both | Only Mobility Service | -2.2049 | 0.013769 | -0.10175 | 0.036143 | * |
| FreqHelp | Both | None | -2.98106 | 0.000867 | -0.12465 | 0.00347 | ** |
| FreqHelp | Only Tactile Map | Only Mobility Service | 1.797081 | 0.044683 | 0.117391 | 0.091545 | n.s. |
| FreqHelp | Only Tactile Map | None | 1.506995 | 0.092267 | 0.09529 | 0.164903 | n.s. |
| FreqHelp | Only Mobility Service | None | -0.76629 | 0.391959 | -0.02336 | 0.498856 | n.s. |
|  | | | | | | | |
| OMOscore | Both | Only Tactile Map | 4.45719 | 7.95E-06 | 0.279558 | 9.54E-05 | *** |
| OMOscore | Both | Only Mobility Service | 2.02444 | 0.042492 | 0.131646 | 0.089233 | n.s. |
| OMOscore | Both | None | 6.261368 | 3.51E-10 | 0.247602 | 9.82E-09 | *** |
| OMOscore | Only Tactile Map | Only Mobility Service | -3.44568 | 0.000555 | -0.21906 | 0.002588 | ** |
| OMOscore | Only Tactile Map | None | -0.90748 | 0.36315 | -0.06674 | 0.492009 | n.s. |
| OMOscore | Only Mobility Service | None | 5.553181 | 2.62E-08 | 0.176321 | 5.51E-07 | *** |
|  | | | | | | | |
| Education | Both | Only Tactile Map | 0.076961 | 0.93667 | -0.00114 | 0.947955 | n.s. |
| Education | Both | Only Mobility Service | 2.062027 | 0.033263 | 0.102497 | 0.073778 | n.s. |
| Education | Both | None | 4.092604 | 2.39E-05 | 0.167982 | 0.000251 | *** |
| Education | Only Tactile Map | Only Mobility Service | 1.364048 | 0.159048 | 0.094649 | 0.252077 | n.s. |
| Education | Only Tactile Map | None | 2.645253 | 0.006314 | 0.161739 | 0.021214 | * |
| Education | Only Mobility Service | None | 2.511989 | 0.009502 | 0.078128 | 0.02884 | * |
|  | | | | | | | |
| EconomicClass | Both | Only Tactile Map | 1.657371 | 0.050227 | 0.118612 | 0.100454 | n.s. |
| EconomicClass | Both | Only Mobility Service | 1.530404 | 0.070603 | 0.069636 | 0.131792 | n.s. |
| EconomicClass | Both | None | 2.966382 | 0.000457 | 0.124094 | 0.00226 | ** |
| EconomicClass | Only Tactile Map | Only Mobility Service | -0.73514 | 0.385119 | -0.04799 | 0.497693 | n.s. |
| EconomicClass | Only Tactile Map | None | 0.088723 | 0.91652 | 0.00558 | 0.938874 | n.s. |
| EconomicClass | Only Mobility Service | None | 1.766544 | 0.036888 | 0.053487 | 0.079451 | n.s. |

**5. Cochrane Q tests to investigate frequency of answers in questions where participants could select more than one option.**

**Table S18: General sample, context of tactile maps usage**

**
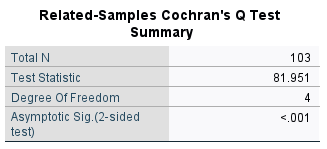
**

**Table S19: General sample, context of tactile maps usage (post-hoc Dunn tests)**

**
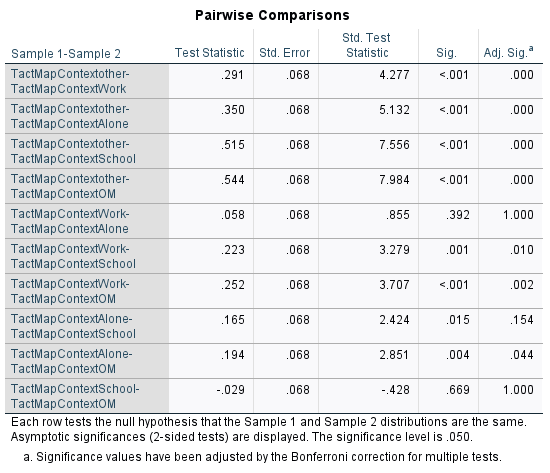
**

**Table S20: General sample, tactile maps methods of production**

**
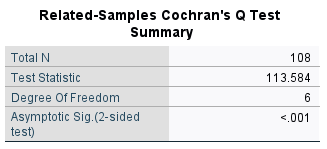
**

**Table S21: General sample, tactile maps methods of production (post-hoc Dunn tests)**

| **Pairwise Comparisons** | | | | | |
| --- | --- | --- | --- | --- | --- |
| Sample 1-Sample 2 | Test Statistic | Std. Error | Std. Test Statistic | Sig. | Adj. Sig.^a^ |
| FabricationMethodPaperBraille-FabricationMethodPaperTexture | -.111 | .059 | -1.869 | .062 | 1.000 |
| FabricationMethodPaperBraille-FabricationMethodHandmadeGLUED | -.148 | .059 | -2.492 | .013 | .267 |
| FabricationMethodPaperBraille-FabricationMethodMultipleMaterial | -.278 | .059 | -4.672 | <.001 | .000 |
| FabricationMethodPaperBraille-FabricationMethodHandmadeVELCRO | -.370 | .059 | -6.229 | <.001 | .000 |
| FabricationMethodPaperBraille-FabricationMethodMetal | -.380 | .059 | -6.385 | <.001 | .000 |
| FabricationMethodPaperBraille-FabricationMethodother | -.528 | .059 | -8.876 | <.001 | .000 |
| FabricationMethodPaperTexture-FabricationMethodHandmadeGLUED | -.037 | .059 | -.623 | .533 | 1.000 |
| FabricationMethodPaperTexture-FabricationMethodMultipleMaterial | -.167 | .059 | -2.803 | .005 | .106 |
| FabricationMethodPaperTexture-FabricationMethodHandmadeVELCRO | -.259 | .059 | -4.360 | <.001 | .000 |
| FabricationMethodPaperTexture-FabricationMethodMetal | -.269 | .059 | -4.516 | <.001 | .000 |
| FabricationMethodPaperTexture-FabricationMethodother | -.417 | .059 | -7.008 | <.001 | .000 |
| FabricationMethodHandmadeGLUED-FabricationMethodMultipleMaterial | -.130 | .059 | -2.180 | .029 | .614 |
| FabricationMethodHandmadeGLUED-FabricationMethodHandmadeVELCRO | -.222 | .059 | -3.737 | <.001 | .004 |
| FabricationMethodHandmadeGLUED-FabricationMethodMetal | -.231 | .059 | -3.893 | <.001 | .002 |
| FabricationMethodHandmadeGLUED-FabricationMethodother | -.380 | .059 | -6.385 | <.001 | .000 |
| FabricationMethodMultipleMaterial-FabricationMethodHandmadeVELCRO | .093 | .059 | 1.557 | .119 | 1.000 |
| FabricationMethodMultipleMaterial-FabricationMethodMetal | .102 | .059 | 1.713 | .087 | 1.000 |
| FabricationMethodMultipleMaterial-FabricationMethodother | -.250 | .059 | -4.205 | <.001 | .001 |
| FabricationMethodHandmadeVELCRO-FabricationMethodMetal | -.009 | .059 | -.156 | .876 | 1.000 |
| FabricationMethodHandmadeVELCRO-FabricationMethodother | -.157 | .059 | -2.647 | .008 | .170 |
| FabricationMethodMetal-FabricationMethodother | -.148 | .059 | -2.492 | .013 | .267 |
| Each row tests the null hypothesis that the Sample 1 and Sample 2 distributions are the same.  Asymptotic significances (2-sided tests) are displayed. The significance level is .050. | | | | | |
| a. Significance values have been adjusted by the Bonferroni correction for multiple tests. | | | | | |

**Table S22: General sample, categories of environments represented by tactile maps**

**
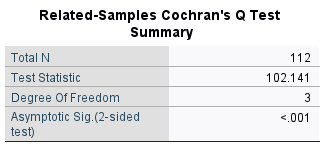
**

**Table S22: General sample, categories of environments represented by tactile maps (Post-hoc Dunn tests)**

**
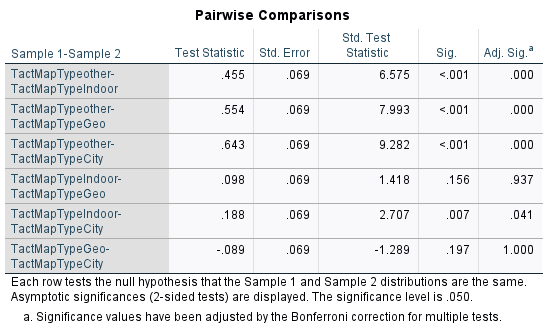
**

**Table S23: General sample, goal of tactile map usage**

**
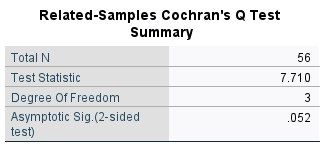
**
